# Synovial tissue atlas in juvenile idiopathic arthritis reveals pathogenic niches associated with disease severity

**DOI:** 10.1126/scitranslmed.adt6050

**Published:** 2025-07-02

**Authors:** Chrissy Bolton, Christopher B. Mahony, Elizabeth Clay, Patricia Reis Nisa, Søren Lomholt, Annie Hackland, Paulynn S. Chin, Charlotte G. Smith, Vicky Alexiou, Huong D. Nguyen, Manigandan Thyagarajan, Zishan Sheikh, Penny Davis, Samantha Chippington, Sandrine Compeyrot-Lacassagne, Sunit Davda, Charlene Foley, Inga Turtsevich, Benjamin Ingledow, Klaudia Kupiec, James Kelly, Megan M. Hanlon, Edward DiCarlo, Luke J. Jones, Samantha L. Smith, Stephen Eyre, Georgiana Neag, Samuel Kemble, Roopa Madhu, Mukta G. Palshikar, Ilya Korsunsky, Ce Gao, Miles Tran, Calliope Dendrou, Christopher D. Buckley, Mark C. Coles, Karim Raza, the MAPJAG Study Group - the following are collaborators who have contributed towards patient care, sample acquisition, study design, or data interpretation, Holly R. Adams, Hussein Al-Mossawi, Rehana Begum, Ian Beh, Catherine Cotter, Jenny Crook, Serena Cruickshank-Hull, Nia Evans, Lisa Fuller, Genevieve Gottschalk, Nadege Haouidji-Javaux, Ruth Howman, Kimme Hyrich, Maryam Imran, Persephone Jenkins, Kelsey Jones, Saffron King, Kerry Leslie, Neelam Khan, Brian Marsden, Lucy-Jayne Marsh, Diarmuid McLaughlin, Alyssia McNeece, Rafeeq Muhammed, Chadwick Pils, Emily Powell, Sugrah Sultan, Laura Threadgold, Holm Uhlig, Qiong Wu, Ellen Gravallese, Andrew Filer, Kevin Wei, Eslam Al-Abadi, Elizabeth C. Rosser, Lucy R. Wedderburn, Adam P. Croft

**Affiliations:** 20 Nuffield Department of Orthopaedics, Rheumatology and Musculoskeletal Sciences, University of Oxford; 1 Infection, Immunity and Inflammation Research and Teaching Department, University College London (UCL) Great Ormond Street Institute of Child Health; London, UK, WC1N 1EH; 2 Department of Inflammation and Ageing, University of Birmingham; Birmingham, UK, B15 2TT; 3 Kennedy Institute of Rheumatology, University of Oxford; Oxford, UK, OX3 7FY; 4 Department of Biomedicine, Aarhus University; Aarhus, Denmark, DK-8000; 5 Department of Pediatric and Adolescent Medicine, Aarhus University Hospital; Aarhus, Denmark, DK-8200; 6 Department of Ageing, Rheumatology and Regenerative Medicine, Division of Medicine, UCL; London, UK, WC1E 6JF; 7 Centre for Adolescent Rheumatology Versus Arthritis at UCL, UCL Hospitals and Great Ormond Street Hospital (GOSH); London, UK, WC1E 6JF; 8 NIHR Biomedical Research Centre at Great Ormond Street Hospital NHS Foundation Trust; London, UK, WC1N 3JH; 9 Birmingham Women’s and Children’s Hospital NHS Foundation Trust; Birmingham, UK, B15 2TG; 10 Department Interventional Radiology, Great Ormond Street Hospital NHS Foundation Trust; London, UK, WC1N 3JH; 11 Department of Rheumatology Great Ormond Street Hospital NHS Foundation Trust; London, UK, WC1N 3JH; 12 Division of Rheumatology, Inflammation, and Immunity, Department of Medicine, Brigham and Women’s Hospital and Harvard Medical School; Boston, MA, USA, 02115; 13 Department of Pathology and Laboratory Medicine, Hospital for Special Surgery; New York, NY, USA, 10021; 14 Centre for Genetics and Genomics Versus Arthritis, Centre for Musculoskeletal Research, Manchester Academic Health Science Centre, University of Manchester; Manchester, UK, M13 9PT; 15 National Institute for Health and Care Research (NIHR) Manchester Biomedical Research Centre, Manchester University NHS Foundation Trust, Manchester Academic Health Science Centre; Manchester, UK, M13 9WU; 16 Division of Genetics, Department of Medicine, Brigham and Women’s Hospital and Harvard Medical School; Boston, MA, USA, 02115; 17 Broad Institute of Massachusetts Institute of Technology; Cambridge, MA, USA, 02142; 18 National Institute for Health and Care Research (NIHR) Oxford Biomedical Research Centre; Oxford, UK, OX3 9DU; 19 National Institute for Health and Care Research (NIHR) Birmingham Biomedical Research Centre; Birmingham, UK, B15 2TH

## Abstract

Precision application of targeted therapies is urgently needed to improve long-term clinical outcomes for children affected by inflammatory arthritis, known as Juvenile Idiopathic Arthritis (JIA). Progress has been hampered by our limited understanding of the cellular basis of inflammation in the target tissue of the disease, the synovial membrane. Here, we analyzed biopsies from the inflamed joints of treatment-naïve children with JIA, early in the course of their disease, using single-cell RNA sequencing, multiplexed immunofluorescence, and spatial transcriptomics to establish a cellular atlas of the JIA synovium. We identified distinct spatial tissue niches, composed of specific stromal and immune cell populations. Additionally, we localized genes linked to arthritis severity and disease risk to effector cell populations, including tissue resident *SPP1+* macrophages and fibrin-associated myeloid cells. Combined analyses of synovial fluid and peripheral blood from matched individuals revealed differences in cellular composition, signaling pathways and transcriptional programs across these distinct anatomical compartments. Furthermore, our analysis revealed several pathogenic cell populations that are shared with adult-onset inflammatory arthritis, as well as age-associated differences in tissue vascularity, prominence of innate immunity and enrichment of TGF-β-responsive stromal subsets that upregulate expression of disease risk-associated genes. Overall, our findings demonstrate the need for age-specific analyses of synovial tissue pathology to guide targeted treatment strategies in JIA.

## Introduction

Juvenile idiopathic arthritis (JIA) is the most common inflammatory rheumatic disease in childhood, affecting more than two million children worldwide (1). One to two thirds of children with JIA experience arthritis that persists into adulthood, leading to long-term joint damage and disability (2). The underlying pathophysiology and molecular drivers of JIA are poorly understood and despite the therapeutic advances of the last two decades, long-term outcomes for children with JIA remain disappointing (3). Response to targeted therapies are highly variable, with no reliable predictors of drug response or disease severity (4). The current trial-and-error approach to treatment selection carries a high risk of failure, often meaning children are treated with multiple medications over the life-course of their arthritis, which can lead to poor long-term disease outcomes (4).

High-resolution analysis of the synovial tissue from inflamed joints in adults, using single-cell profiling technologies, has uncovered the cellular heterogeneity of the arthritic synovium. This approach has elucidated disease-associated cell states, new tissue-resident cell subsets, and key cellular interactions underpinning active disease and remission (5, 6). In JIA, practical challenges have limited our ability to study the synovium directly, leading to a reliance on surrogate molecular signals detectable in blood and synovial fluid (7). However, this approach has not yielded any validated biomarkers of treatment response or disease trajectory, and the correlation between the cellular and molecular pathology in the blood and synovium is poorly understood (7).

In the present study, we applied minimally invasive, ultrasound-guided tissue biopsies to sample the synovium in the early phases of JIA. Using single-cell next-generation sequencing technologies, we define a cellular atlas of the inflamed synovium in childhood-onset inflammatory arthritis. We demonstrate how immune and stromal cell populations are organized into spatially distinct niches within the inflamed synovium and describe age-associated differences in cellular composition that may impact response to specific targeted therapies. Finally, we mapped genes relevant to disease, including those associated with severity, to effector cell populations in the synovium.

## Results

### Multimodal single-cell profiling of synovial tissue, synovial fluid, and blood in children with JIA

**Figure 1:**
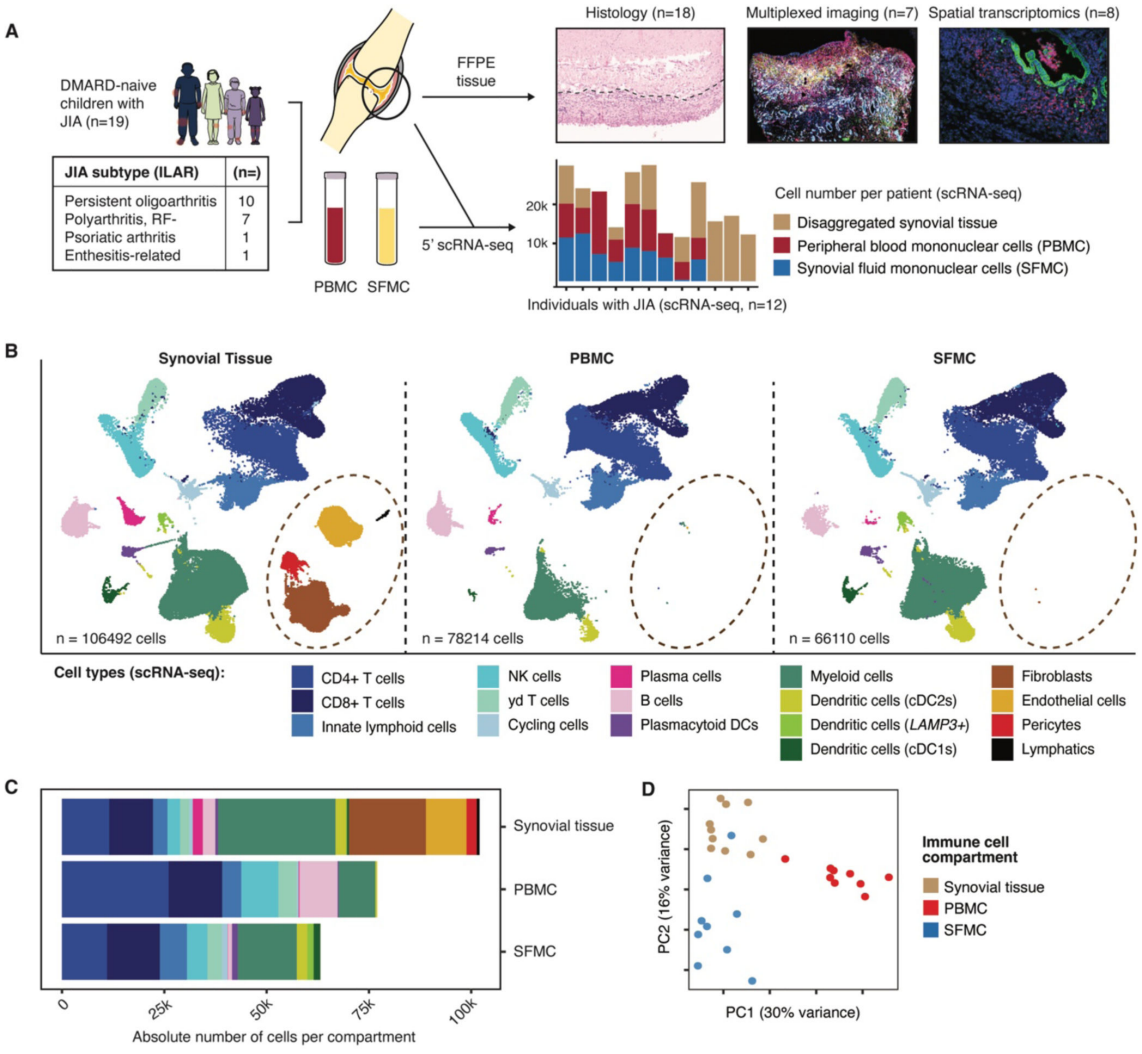
Synovial tissue biopsies from children with JIA capture specific cell populations not represented in blood or synovial fluid. (**A**) Schematic showing study overview. Minimally invasive tissue biopsies with matched synovial fluid mononuclear cells (SFMC) and peripheral blood mononuclear cell (PBMC) samples were collected from children with JIA who were DMARD-naïve. Table shows JIA subtypes included in analysis. ILAR, International League of Associations for Rheumatology; RF, rheumatoid factor. Fresh frozen paraffin embedded (FFPE) biopsy samples were analyzed by histology (*n* = 18), multiplexed immunofluorescence imaging (Leica Cell DIVE, *n* = 7), and spatial transcriptomics (10x Genomics Xenium, *n* = 8). Synovial tissue biopsies and PBMC and SFMC were analyzed by scRNA-seq (10x Genomics Chromium), cell number per patient and anatomical compartment are presented as stacked bar graph (*n* = 12). Portions of the schematic are adapted from Servier Medical Art graphics, licensed under CC BY 4.0. (**B**) Seurat-integrated Uniform Manifold Approximation and Projection (UMAP) embeddings of main cell type annotations split by anatomical compartment and colored by cell type. Synovial tissue, *n* = 10; PBMC, *n* = 9; SFMC, *n = 9*. (**C**) Total number of synovial tissue cells, PBMC and SFMC analyzed by scRNA-seq following quality control. Colors indicate cell type from (B). (**D**) PCA of immune cell type abundance from scRNA-seq. Dots indicate individual participant samples of different specimen types.

To determine the cellular architecture of the inflamed synovium in JIA, 19 children underwent biopsies of their joint synovia, a median of 19 days after clinical diagnosis (Fig. 1A, table S1). Peripheral blood and synovial fluid samples from the same individuals were collected in parallel with tissue biopsies. All children were naïve to treatment with disease-modifying anti-rheumatic drugs (DMARD) for their arthritis at the time of sample collection. The cohort included children with four clinical subtypes of JIA (International League of Associations for Rheumatology classification (ILAR)), with the majority classified as oligoarticular or rheumatoid factor-negative polyarticular JIA (Fig. 1A, table S1). Biopsy samples underwent histopathological assessment and samples from 10 participants were disaggregated for 5’ single-cell RNA-sequencing (scRNA-seq; 10x Genomics Chromium). Spatial profiling technologies, including transcriptomics and multiplexed immunofluorescence, were performed on synovial tissue biopsy samples from 16 individuals. Furthermore, matched samples of peripheral blood mononuclear cells (PBMC) and synovial fluid mononuclear cells (SFMC) from 9 individuals were assessed by 5’ scRNA-seq with paired cellular indexing of transcriptomes and epitopes (CITE-seq) for detection of cell surface antigens (Fig. 1A, fig. S1A).

For the scRNA-seq dataset, after quality control, we clustered and profiled a total of 250,816 cells from synovial tissue, PBMC, and SFMC samples with single-cell resolution (Fig. 1B, fig. S1, A to C). The major cell types that were observed across anatomical compartments were annotated based on gene and cell surface antigen expression (fig. S1A, table S2). In synovial tissue, stromal cell populations included fibroblasts, pericytes, vascular endothelial cells and a small lymphatic endothelial cell population (Fig. 1, B and C). These cell types were absent from the other specimen types, except for a small group of endothelial cells in the PBMC samples and fibroblasts in the SFMC samples (Fig. 1, B and C). Principal component analysis (PCA) of immune cell proportions separated samples by specimen type, indicating a distinct cellular composition of synovial tissue compared with PBMC or SFMC samples (Fig. 1D).

### Myeloid and plasma cell enrichment distinguishes the immune cell composition of synovial tissue from synovial fluid and peripheral blood

**Figure 2:**
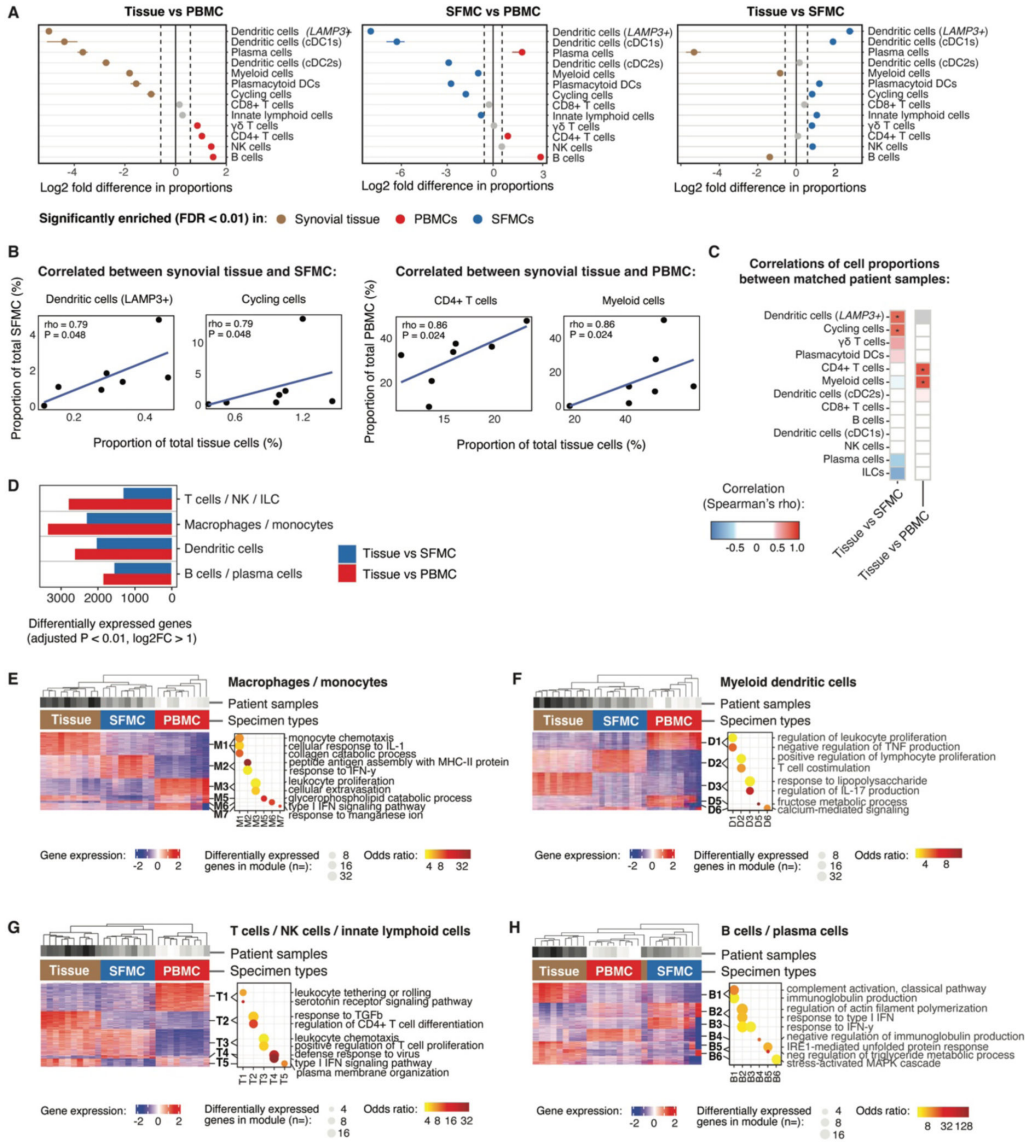
Synovial tissue immune cells differ in phenotype and composition compared with those in synovial fluid or peripheral blood. (**A**) Comparison of proportions of immune cell populations annotated by scRNA-seq data between compartments. Synovial tissue, *n* = 10; SFMC, *n* = 9; and PBMC, *n* = 9. Data are presented as log2 fold change between anatomical compartments. Synovial tissue, brown; PBMC, red; and SFMC, blue. Analysis by Fisher’s exact test with 10,000 permutations. Significantly enriched populations were > 0.58 log2 fold change, FDR < 0.01. Non-significant comparisons shown in grey. (**B**) Scatter plots of significantly correlated immune cell populations between matched specimen types. Dots represent individual participants. Spearman’s correlation analysis. Spearman’s rank coefficient |r| > 0.5, unadjusted *P* < 0.05. *n* = 7. (**C**) Summary heatmap of correlations of immune cell populations between specimen types in matched samples. *n* = 7. Color scale, Spearman’s rank correlation coefficient; *unadjusted *P* < 0.05. (**D**) Bar chart showing number of differentially expressed genes between synovial tissue and SFMC or PBMC samples across the main immune cell lineages (>1 log2 fold change, adjusted *P* < 0.01, DESeq package). (**E to H**) Analysis of differentially expressed gene modules in immune cell lineages between anatomical compartments for macrophages/monocytes (E), myeloid DC (F), T cells/NK cells/ILC (G) and B/plasma cells (H). For each cell lineage, the heatmap (left panel) shows the genes differentially expressed between specimen type; rows represent genes, columns represent participant samples, modules defined by hierarchical clustering. Dot plot (right panel) shows biological pathways (GO terms) associated with upregulated genes in modules, differentially expressed between specimen type (adjusted *P* < 0.05). In (D to H) synovial tissue, *n* = 10; SFMC, *n* = 9; and PBMC, *n* = 9.

To determine how the immune cell composition differed in tissue, we compared the proportions of immune cell lineages across the three anatomical compartments (Fig. 2A). Compared with PBMC samples, proportions of dendritic cells (DC), myeloid cells and cycling cells were enriched in both synovial tissue and SFMC. Plasma cells were significantly more abundant in synovial tissue than SFMC or PBMC (>3.5 log2 fold change (FC), ***P*** < 0.0001). In contrast, DC, plasmacytoid DC (pDC), innate lymphoid cells (ILC), natural killer (NK) cells and gamma-delta T cells were more abundant in SFMC than in synovial tissue.

We next tested whether cellular proportions in synovial fluid or peripheral blood reflected the cellular composition of synovial tissue, at the level of individual participant samples. Cell type abundance between participant-matched samples of tissue and SFMC or PBMC were not correlated for most cell types; however, *LAMP3+* DC and cycling cells in tissue were correlated with SFMC proportions, and CD4+ T cells and myeloid cells in tissue were correlated with PBMC proportions (***P*** < 0.05) (Fig. 2, B and C).

### Gene expression programs in immune cells are dependent on the anatomical location

To examine whether cell types shared across these three compartments displayed location-specific programs of gene expression, we investigated which genes were differentially expressed between specimen types (Fig. 2D, table S3). T cells, myeloid cells, and B cells differentially expressed >1300 genes in synovial tissue compared with SFMC or PBMC. Distinct transcriptional programs associated with varied signaling and effector pathways were differentially enriched in cells across the three anatomical compartments (Fig. 2, E to H, table S4).

In synovial tissue, monocytes and macrophages upregulated pathways associated with pro-inflammatory responses to IL-1 and collagen catabolism, necessary for tissue remodeling (M1 module, Fig. 2E). Response to interferon-γ (IFN-γ) and type-I interferon were upregulated in monocytes and macrophages from SFMC (M2 and M6 modules, Fig. 2E), whereas genes associated with leukocyte proliferation and extravasation were upregulated in PBMC (M3 module). Myeloid DC in tissue displayed a pro-inflammatory phenotype, upregulating lipopolysaccharide-response genes (D3 module, Fig. 2F). Myeloid DC from SFMC were also highly activated, upregulating pathways involved in T cell co-stimulation (D2 module), perhaps contributing to the high proportion of cycling cells in SFMC.

T cells in synovial tissue exhibited increased expression of transforming growth factor-β (TGF-β) response pathways and genes associated with CD4+ T cell differentiation (T2 module, Fig. 2G). In T cells from SFMC, type-I interferon response genes were also upregulated (T4 module), along with genes associated with chemotaxis and proliferation (T3 module). Pathways relating to leukocyte rolling and tethering were upregulated in T cells from PBMC (T1 module). Tissue-enriched B and plasma cells upregulated genes involved in complement activation and immunoglobulin production (B1 module, Fig. 2H). Like other SFMC lineages, type-I interferon and IFN-γ response genes were upregulated in B cells (B2 and B3 modules respectively), along with genes involved in actin filament polymerization (B2 module). The upregulation of interferon response genes in immune cells from SFMC is consistent with the proportional enrichment of pDC and NK cells (key producers of type-I IFNs and IFN-γ) in SFMC compared with synovial tissue. Increased expression of genes in pathways associated with chemotaxis and actin filament polymerization in T and B cells from SFMC may reflect increased motility of the lymphocytes migrating into the synovial fluid. Overall, these findings suggest synovial fluid serves as a reservoir of activated cells and exhibits distinct features of immune dysregulation compared with synovial tissue. These data highlight the limitations of relying on SFMC to infer the cellular pathology of the inflamed synovium in JIA.

### Joint inflammation drives a distinct shift in immune cell composition of the synovium

**Figure 3:**
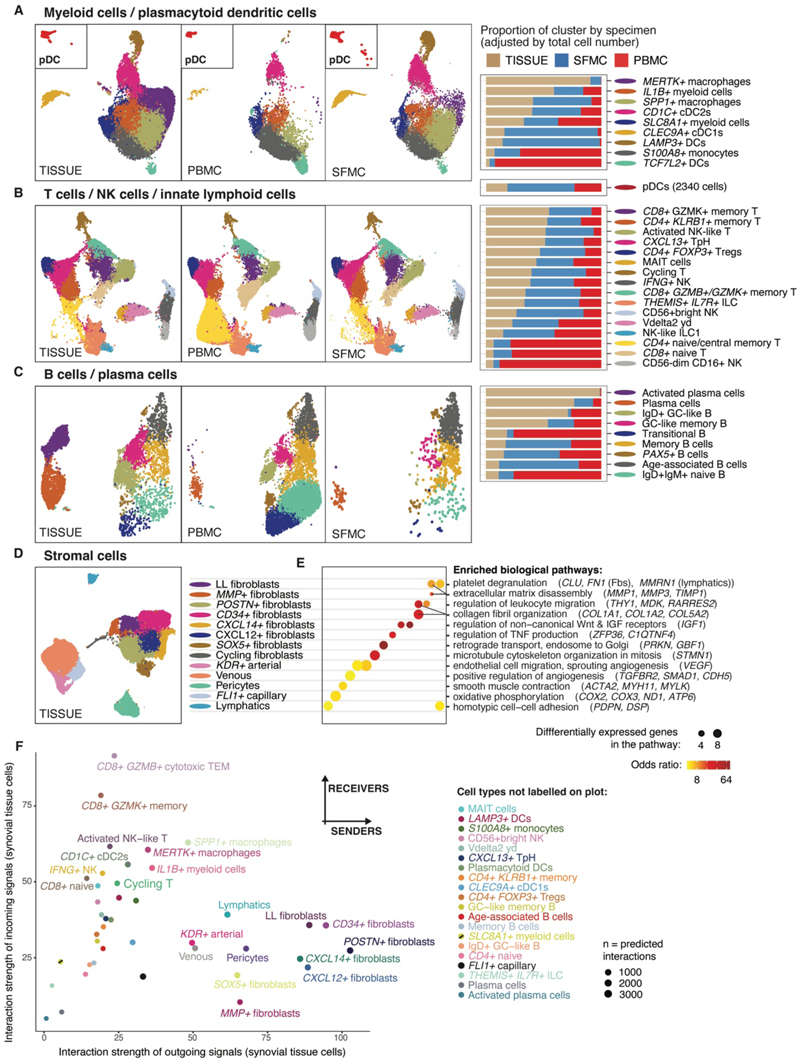
Immune and stromal cell landscape of the inflamed synovium in JIA. (**A to C**) High resolution UMAP clustering of all cells across anatomical compartments for myeloid and plasmacytoid dendritic cells (A); T cells, NK cells, and innate lymphoid cells (B); and B cells and plasma cells (C). Synovial tissue (left), PBMC (middle), and SFMC (right). Clusters are colored according to bar chart labels. Right panel: Bar charts of the contribution of each compartment to cell numbers per cluster. Proportions per specimen type shown are stacked and scaled to 100%. Synovial tissue, *n* = 10; PBMC, *n* = 9; and SFMC, *n* = 9. (**D**) UMAP showing high resolution clustering of stromal cell types in synovial tissue, *n* = 10. (**E**) Analysis of biological pathways (GO terms, adjusted *P* < 0.05) and associated differentially expressed marker genes (>1 log2 fold change) between stromal clusters. Dot size, number of genes in pathways; color scale, odds ratio. (**F**) Scatter plot of predicted interactions between fine cell states in synovial tissue based on aggregate expression of ligand and receptor genes showing interaction strength of total outgoing and incoming signals (CellChat package), unadjusted *P* < 0.05, *n* = 10. Only clusters containing > 300 cells in tissue are visualized; cell clusters not labelled on plot are defined on right hand panel.

To identify how the cellular landscape changes in synovial tissue during arthritis, we compared our data to a published scRNA-seq dataset of non-arthritic adult knee biopsies obtained from bone tumor resections and organ donors (fig. S2, A and B) (8). On average, immune cells made up 70% (± 9%) of cells from the JIA synovial tissue, whereas non-arthritic biopsies were predominantly stromal, with immune cells comprising only 21% (± 3%) of the total cells (*P* < 0.001, fig. S2A). Among major cell types, B cells, plasma cells, and pDC were considerably more abundant in JIA samples, being nearly absent from non-arthritic biopsies (combined total *n* = 43 / 34557 cells) (fig. S2, A and B). To further delineate disease-associated immune cell states, we subclustered JIA synovial tissue, SFMC, and PBMC scRNA-seq data more finely, yielding five myeloid, four DC, nine T cell, five NK cell/ILC and nine B/plasma cell states, described on the basis of gene and, where possible, cell surface antigen expression (Fig. 3, A to C, fig. S3, A to F, table S5). Comparable cell states were identified in adult non-arthritic synovial tissue data using label transfer to assess disease-associated changes in cell type abundance (fig. S4, A to D).

In the myeloid cell fraction, the most tissue-enriched populations in samples from children with JIA included tissue resident *MERTK*+ macrophages and *IL1B+* myeloid cells (Fig. 3A). *MERTK*+ macrophages were enriched in non-arthritic biopsies, suggesting a potentially homeostatic role within the joint (Fig. 3A, fig. S4, A and B). Within the JIA cohort, PBMC samples contributed the largest proportion of *S100A8+* monocytes, which had high *FCN1* expression, and *TCF7L2+* DC, suggesting these primarily infiltrate the tissue from a circulating reservoir (Fig. 3A). *SPP1+* macrophages with high expression of lining layer markers fibronectin and *TREM2* (9, 10) and the chemoattractant *CXCL10* were the most abundant myeloid cells in SFMC (Fig. 3A, fig. S3A). An *SLC8A1*+ myeloid cluster expressing *MERTK* and *ID2* resembled the proposed homeostatic *MERTK*+ precursors (6, 10) (fig. S3A). This population was significantly enriched in JIA tissue compared with the non-arthritic synovium (FDR < 0.01, fig. S4, A and B). Dendritic cells were distinguished from monocytes by their lower surface protein levels of complement receptor CR1, associated with phagocytosis (fig. S3B). Of these, conventional type 2 DC (*CD1C+* cDC2) were the most abundant DC population in JIA tissue and strongly expressed the activation marker *NR4A1 (*Fig. 3A, fig. S3, A and B). Rarer conventional type 1 DC (*CLEC9A+XCR1+* cDC1) and DC expressing the maturation marker *LAMP3* present in SFMC and JIA tissue were almost undetectable in PBMC samples and non-arthritic tissue (Fig. 3A, fig. S4, A and B). In the JIA cohort, multiple myeloid subclusters, including *IL1B+* and *SLC8A1*+ myeloid cells were positively correlated with age, making up a larger proportion of cells in older children (fig. S5A). This correlation could not be explained by JIA disease duration (fig. S5B).

### Diverse unconventional T cell and memory T cell states dominate the inflamed synovial tissue

T cell subclusters had a similar distribution between JIA synovial fluid and tissue compartments, once adjusted for the total cell number in each specimen type. As expected, in JIA samples, CD45RO+ memory T cells were enriched in both synovial tissue and SFMC, whereas CD45RA+ naïve T cells were enriched in PBMC (Fig. 3B, fig. S3C). *CD8+GZMK+* memory T cells and *CD4+KLRB1+* memory T cells were the most enriched T cell populations in JIA tissue (Fig. 3B). Only a very small proportion of activated NK-like T cells originated from JIA PBMC samples or were detectable in non-arthritic adult tissue (Fig. 3B, fig. S4C). This mixed *XCL1*+*ZNF683+* cluster of *CD8+* T cells and type 1 gamma-delta T cells (VD1+ γδ) had high expression of NK markers *GNLY* and *KLRC2*, but low expression of cytotoxic molecules such as granzymes and perforin (fig. S3A). Consistent with recent studies (11), this population incorporating VD1+ γδ T cells was more enriched in younger children, showing a negative correlation with age (fig. S5A). To identify cells with a resident memory T cell phenotype, we scored cells for their combined expression of reported tissue-residency markers *ITGAE* (CD103), *ITGA1* (CD49a), *PDCD1* (PD-1) and *CXCR6* (12) (fig. S3D). Cells with the highest expression of these markers localized to the *CXCL13+* T peripheral helper cluster, which contained both CD4+ and CD8+ cells, and the *CD8+ GZMB+ / GZMK+* memory T cell cluster. There was an absence of cells highly expressing these markers in PBMC samples (fig. S3D).

The presence or absence of αβ-T cell receptors clearly delineated conventional and unconventional T cells and confirmed our manual annotation of NK cells, ILC, and gamma-delta T cells (fig. S3E). Recent studies have identified a ‘MT-high’ T cell population with a high amount of mitochondrial RNA (13), which we identified as ILC in our dataset (NK-like ILC1 and *THEMIS*+ *IL7R*+ ILC). This SFMC-enriched group of cells exhibited high expression of CD3z (*CD247*) and *MTRNR2L12*, as well as key transcription factors (*TOX, BCL11B* and *RORA*) (14) (fig. S3A). Among NK cells, *IFNG+(ITGAE+)* NK cells were the most tissue-enriched in JIA samples, whereas CD56^dim^CD16+ NK cells were more enriched in JIA PBMCs and in non-arthritic tissue samples compared with JIA synovial tissue (Fig. 3B, fig. S4C).

### B cells within the synovial tissue display germinal center-like states

IgG+ plasma cells and activated IgG+(*JUN+*) plasma cells were highly enriched in JIA tissue and more abundant in younger children within this cohort (Fig. 3C, fig. S5A). The enrichment of plasma cells was not exclusive to children with oligoarticular disease nor observed in all children with anti-nuclear antibody seropositivity. Compared to PBMC/SFMC samples and non-arthritic biopsies, naïve and memory B cells expressing germinal center (GC) markers such as *BCL2A1* and *CD83* were enriched in JIA tissue (IgD+ GC-like B and GC-like memory B cells), suggesting the acquisition of this cell state upon entering the inflamed tissue microenvironment (Fig. 3C, fig. S4C). *ITGAX+* age-associated B cells and memory B cells were the most enriched clusters in JIA SFMC and strongly expressed typical type-I IFN-response genes (*MX1, IFITM1;* Fig. 3C, fig. S3, A and F). As expected, naïve IgD+ B cells and (*SOX4+RAG1+)* transitional B cells were more enriched in JIA PBMC than other compartments. A *PAX5*+ B cell cluster was identified that expressed a number of pro-B cell markers (*EBF1, PAX5, BACH2*) (15) and high proportions of mitochondrial RNA (fig. S3A).

### Stromal-derived signals dominate cellular communication in the inflamed synovium

Within the stromal cells in the inflamed JIA synovium, fine sub-clustering yielded eight fibroblast, one pericyte and four vascular subclusters (Fig. 3D). Two clusters, lining layer fibroblasts and *MMP+* fibroblasts, expressed classical lining layer markers (*PRG4, PDPN*), high expression of matrix metalloproteinases, and upregulated extracellular matrix disassembly pathways (Fig. 3E, fig. S3A, table S6). *POSTN+* and *CD34+(MFAP5+)* fibroblasts had the highest expression of collagen 1 (*COL1A1, COL1A2*) and genes associated with collagen fibril organization (Fig. 3E, fig. S3A). *POSTN+* fibroblasts were the most enriched population in JIA tissue compared with non-arthritic biopsies (fig. S4D). *CXCL14*+ fibroblasts highly expressed the proposed universal fibroblast progenitor marker *PI16* (16) and upregulated genes involved in insulin-like growth factor responses and non-canonical Wnt signaling (Fig. 3E, fig. S3A). This population was significantly enriched in non-arthritic biopsies compared with JIA samples, suggesting a non-pathogenic role (FDR < 0.01, fig. S4D). The fibroblast cluster upregulating the chemoattractant *CXCL12* also highly expressed *SFRP1*, an extracellular modulator of the Wnt pathway that influences the acquisition of an invasive fibroblast state (17). Venous cells could be distinguished by their expression of the atypical cytokine receptor *ACKR1*, characteristic of post-capillary venules, and the absence of *NOTCH4* gene expression seen in the arterial and capillary clusters (fig. S3A). The venous cluster also highly expressed *POSTN*, involved in repair and remodeling following injury (18). Arterial cells highly expressed the VEGF-A receptor *KDR* and upregulated pathways that promote angiogenesis (Fig. 3E).

To identify cell-cell interactions at the site of inflammation, we performed an interactome analysis of the ligands and receptors expressed by JIA synovial tissue cells, which suggested that stromal cells produced the strongest signals dictating the microenvironment (Fig. 3F). Specifically, *CD34+* fibroblasts, *POSTN+* fibroblasts, lining layer fibroblasts, and *CXCL12+* fibroblasts were predicted to be the dominant producers of signals in the inflamed joint. Among immune cells, tissue-enriched *MERTK+* macrophages, *IL1B+* myeloid cells, and *SPP1+* macrophages expressed the strongest outgoing signals, whereas *GZMB+* and *GZMK+CD8+* T cells had the greatest predicted response to incoming signals, suggesting they are highly reactive to external stimuli within the microenvironment (Fig. 3F).

### The inflamed synovial tissue is composed of distinct cellular niches

**Figure 4:**
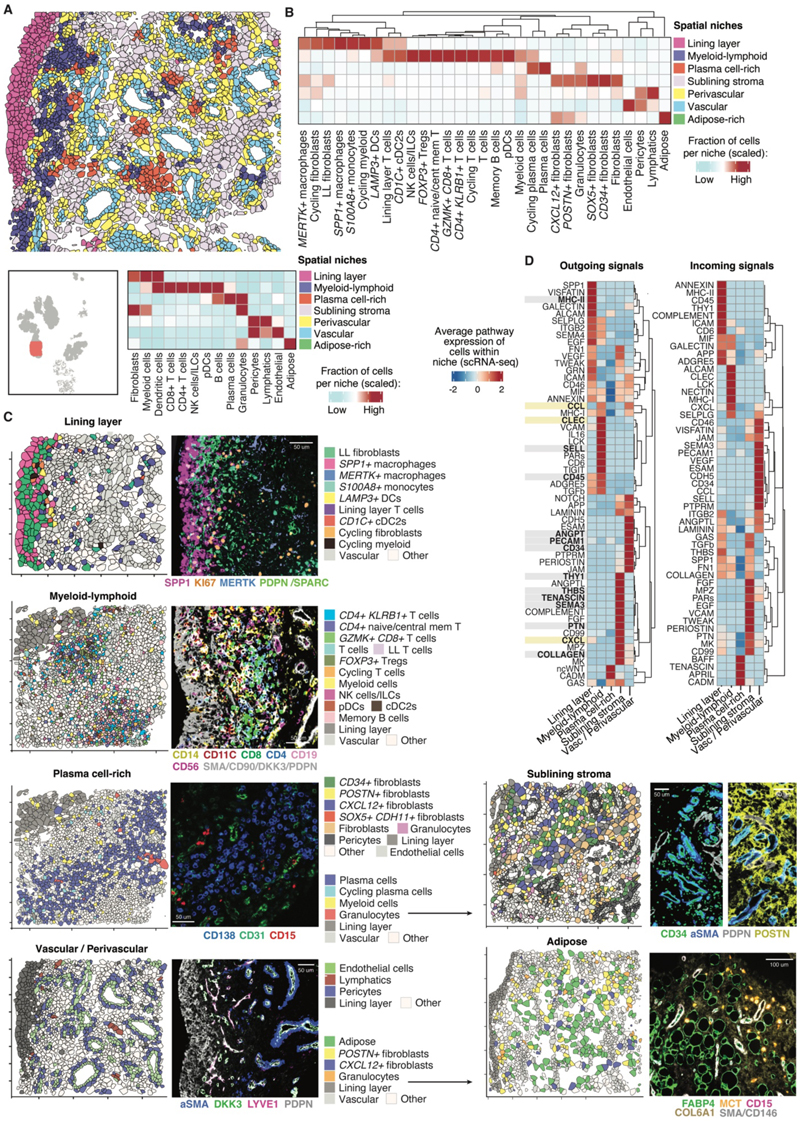
The inflamed synovial tissue architecture in JIA is composed of distinct tissue niches. (**A**) Representative annotation of spatial transcriptomic section showing the distribution of 7 spatial niches (regions of shared gene expression profiles) in a synovial tissue fragment (top). Polygons represent individual cells detected; polygon color represents niche annotation. Region of biopsy section visualized is highlighted in red (bottom left). Heatmap shows cellular composition of the 7 spatial niches identified (bottom right). *n* = 8 children (365,279 cells over 413 fields of view). (**B**) Heatmap showing cellular composition of finely clustered cell states in the 7 spatial niches, grouped by hierarchical clustering. Rows, niche; Columns, cell states as defined in fig. S6, A and B. (**C**) For each spatial niche, a representative section of synovial tissue shows distribution of cell annotations from spatial transcriptomic analysis in each niche (left), multiplexed immunofluorescence of cell protein marker expression (right). Polygon colors, cell annotations in associated key; colors for antibody markers indicated below image (right). Vascular and perivascular niches are shown in the same panel. Spatial transcriptomic plots are not matched to multiplexed immunofluorescence images. Key for adipose and plasma cell-rich niche shown left of images. (**D**) Heatmap showing top predicted interactions of cells comprising each niche, based on aggregate expression of ligand and receptor genes in synovial tissue (scRNA-seq), unadjusted *P* < 0.05; *n* = 10. Unsupervised hierarchical clustering illustrates the inferred communication networks and distinct signaling pathways associated with each niche. For outgoing signals, pathways with genes present in the 377-gene spatial transcriptomics panel are highlighted in bold (grey = highlighted in fig. S6C, yellow = highlighted in fig. S6D). Vasc, vascular.

To further hone our analysis of cellular interactions, we sought to focus on populations that were in close spatial proximity within the tissue microenvironment. We used spatial transcriptomics (10x Genomics Xenium, with the Xenium Human Multi-Tissue and Cancer Gene Expression and hMulti_v1 gene panel) to profile JIA synovial tissues sections, facilitating localization of specific cell states. The spatial expression of 377 genes from 365,279 cells over 413 fields of view were analyzed from 8 JIA synovial tissue samples, with cell identities deconvoluted using our synovial tissue scRNA-seq data (fig. S6, A and B, table S7 and S8). We identified seven transcriptionally distinct niches, based on gene expression, cell type composition, and spatial location (Fig. 4, A to C). This spatial analysis allowed us to focus the ligand-receptor interactions from our scRNA-seq data to those between cells within the same spatial tissue niche (Fig. 4D).

The lining layer niche contained *SPP1+* macrophages interfacing with the synovial cavity, with *PRG4+* lining layer fibroblasts located immediately beneath (Fig. 4, B and C). Multiplexed immunofluorescence confirmed the presence of *SPP1+* macrophages in the lining layer (Fig. 4C). We hypothesized that the abundance of *SPP1+* macrophages in SFMC may therefore arise from these cells being sheared off the surface of the membrane into the synovial fluid. SPP1 interactions were a top predicted outgoing signaling pathway within this niche (Fig. 4D). Within the lining layer niche, we also found *LAMP3*+ DC, *CD1C*+ cDC2, *S100A8*+ monocytes and a small population of T cells (Fig. 4, B and C). *MERTK+* macrophages were enriched in multiple niches and were found to interface with lining layer fibroblasts and vascular cells (Fig. 4, B and C), in keeping with the transcriptional diversity of this cellular subset (6, 10).

In the sublining, we observed a myeloid-lymphoid niche, seen as a dense band on immunofluorescence staining beneath the lining layer containing CD4+ and CD8+ T cell subsets, pDC, myeloid cells, NK cells, and cycling plasma cells (Fig. 4, B and C). Cells within this niche upregulated several adhesion-related pathways including those involving VCAM, P-Selectin (SELPLG) and ALCAM (Fig. 4D). Outgoing TGF-β signaling pathways were also strongly upregulated in this niche (Fig. 4D). A distinct plasma cell-rich niche in the sublining was composed of plasma cells and granulocytes (Fig. 4, B and C). The *BAFF* and *APRIL* pathways, known for their role in B cell survival and maturation (19), were highlighted as key signals influencing this niche (Fig. 4D).

The vascular and perivascular niches encompassed pericytes, lymphatic vessels and endothelial cells, with lymphatics identifiable by immunofluorescence in the superficial sublining from their co-expression of *PDPN* and *LYVE1* (Fig. 4, B and C). Consistent with previous studies of adult-onset arthritis (20), outgoing *NOTCH* signaling was highest in vascular cells (Fig. 4D). Outgoing chemokine signaling (*CCL*) pathways were upregulated by cells that comprised the vascular/perivascular niches and the myeloid-lymphoid niche, emphasizing their role in leukocyte recruitment (Fig. 4D). The sublining stromal niche included granulocytes and all remaining fibroblast subsets (Fig. 4, B and C). *POSTN+* fibroblasts were localized with immunofluorescence to regions around vasculature (Fig. 4C). As expected, collagen-, fibroblast growth factor- (*FGF*) and *THY1*- signaling was upregulated by cells within the stromal sublining niche (Fig. 4D). Finally, an adipose-rich niche was identified containing adipose cells, mast cells, *POSTN+* and *CXCL12+* fibroblasts (Fig. 4, B and C), suggesting a possible role for these fibroblast states in tissue remodeling during inflammation (21). As adipose cells were not present in our scRNA-seq dataset, it was not possible to examine the putative signaling pathways within this niche.

### Cellular signaling networks localize to distinct anatomical niches within the synovial tissue

We next validated the predicted incoming and outgoing signals of each niche (from scRNA-seq ligand-receptor analysis, Fig. 4D) with a specific interrogation of genes found in our spatial transcriptomic dataset (fig. S6, C and D). Increased expression of genes from 12 out of 15 outgoing signaling pathways could be confirmed in the relevant niches of the spatial transcriptomic data, including, for example, increased *SELL* in the myeloid-lymphoid niche and *HLA-DQB2* in the lining layer niche, lending weight to the prediction that trafficking- and MHC class II-related pathways are involved in the myeloid-lymphoid and lining layer niches, respectively (Fig. 4D, fig. S6C). Exceptions to the rule included those genes involved in *CXCL, CLEC* and *CCL* signaling pathways, where distinct cytokines and C-type lectins may have diverse functions and expression profiles, not well-summarized by the single aggregated pathway (fig. S6D).

### Composition of cellular niches within the synovium varies with the severity of inflammation

To assess how the abundance of cells in different spatial niches related to the degree of tissue inflammation, we examined the synovitis scores from histological assessment (22) of sections from the same biopsy tissues. Despite a limited sample size, we observed a positive correlation between the proportion of cells within the plasma cell and myeloid-lymphoid niches and the Krenn inflammatory infiltrate scores (fig. S7A). This suggests that although this measure does not differentiate between distinct immune cell compositions, these niches are associated with severity of joint inflammation. In contrast, niches with a higher degree of stromal cells, including sublining stroma, perivascular and lining layer niches, showed a negative correlation with infiltrate scores (fig. S7A). Accordingly, the proportions of both cycling plasma cells and cycling T cells were associated with higher infiltrate scores on spatial transcriptomic sections, whereas cycling myeloid and cycling fibroblast populations were negatively correlated (fig. S7B).

### Disease-associated biomarkers are enriched in fibrin-infiltrating myeloid cells of the lining layer niche

**Figure 5:**
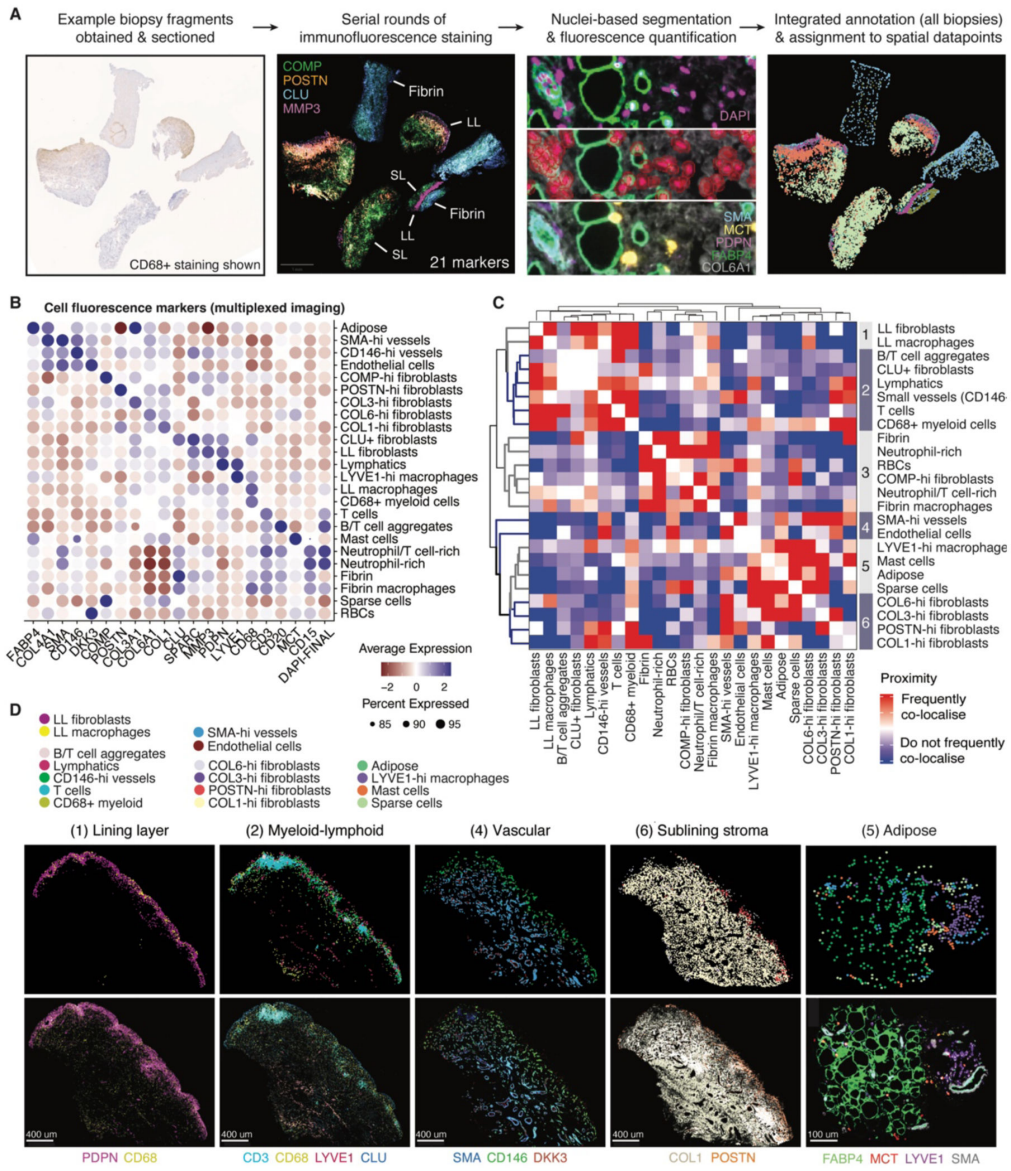
Multiplexed immunofluorescence validates the proximity of cells within spatial tissue niches. (**A**) Analysis workflow for identifying and quantifying cell types using multiplexed immunofluorescence staining to investigate cell localization in spatial niches across multiple biopsies, *n* = 7. Left: representative example of synovial biopsy tissue fragments stained for CD68 expression; middle-left: synovial biopsy tissue following serial rounds of staining with fluorescently-labelled antibodies (LL = lining layer, SL = sublining); middle-right: images demonstrating calculation of marker expression per cell, showing nuclei detection (top) and cell segmentation (middle) to identify individual cells and calculation of marker fluorescence per cell (bottom); right: representative example of cell annotation following integration of data from all biopsy sections. (**B**) Dotplot showing cell type annotation of synovial tissue cell populations visualized by multiplexed immunofluorescence, based on average expression of 21 nuclear and cytoplasmic markers; *n = 7*. Color scale, relative average expression; dot size, percentage of detected cells expressing marker. (**C**) Heatmap showing proximity analysis of cell types in synovial tissue from multiplexed immunofluorescence based on the nearest neighbor of each cell. Hierarchical clustering of proximity scores shows groups of cells that co-localize, samples as in (**B**). Color scale, frequency of co-localization by nearest neighbor. (**D**) Representative multiplexed immunofluorescence images of synovial tissue split by cell groupings identified from proximity analysis. Imaging (bottom panel) and computational annotation of detected cells are shown (top panel, dot color = cell types indicated in key above). Scale bars, 400 µm or 100 µm (adipose).

To validate the spatial localization of cells within the seven defined tissue niches, we analyzed multiplexed immunofluorescence staining of synovial tissue sections (Fig. 5A). Following cell segmentation, the average fluorescence of 21 nuclear and cytoplasmic markers was used to annotate cell types (Fig. 5B, table S9). Hierarchical clustering of proximity scores identified 6 groups of co-localizing cells which corresponded to the niches identified in our spatial transcriptomic analysis (Fig. 5, C and D, Fig. 4, A to C). Compared with the transcriptionally defined niches identified from the spatial transcriptomic data, some additional features of cellular zonation were revealed from multiplexed immunofluorescence. Lymphatics and small CD146-high blood vessels clustered within the myeloid-lymphoid niche in the superficial sublining (group 2 in Fig. 5, C and D). Vessels approximating the lining layer became smaller in diameter and expressed higher levels of CD146 protein, associated with leukocyte trafficking (23), compared with vessels deeper in the sublining expressing more smooth muscle actin (SMA-hi vessels; Fig. 5D, fig. S7C). LYVE1+ macrophages were abundant in the adipose-rich group, potentially reflecting their homeostatic function (10) (Fig. 5, C and D). From immunofluorescence staining, it was apparent that the granulocytes present in the adipose-rich niche in our spatial transcriptomic data corresponded to MCT+ mast cells (Fig. 5D). Accordingly, MCT+ mast cells had greater proximity to collagen-rich fibroblasts and adipose cells than other granulocytes included in the analysis (Fig. 5C).

**Figure 6:**
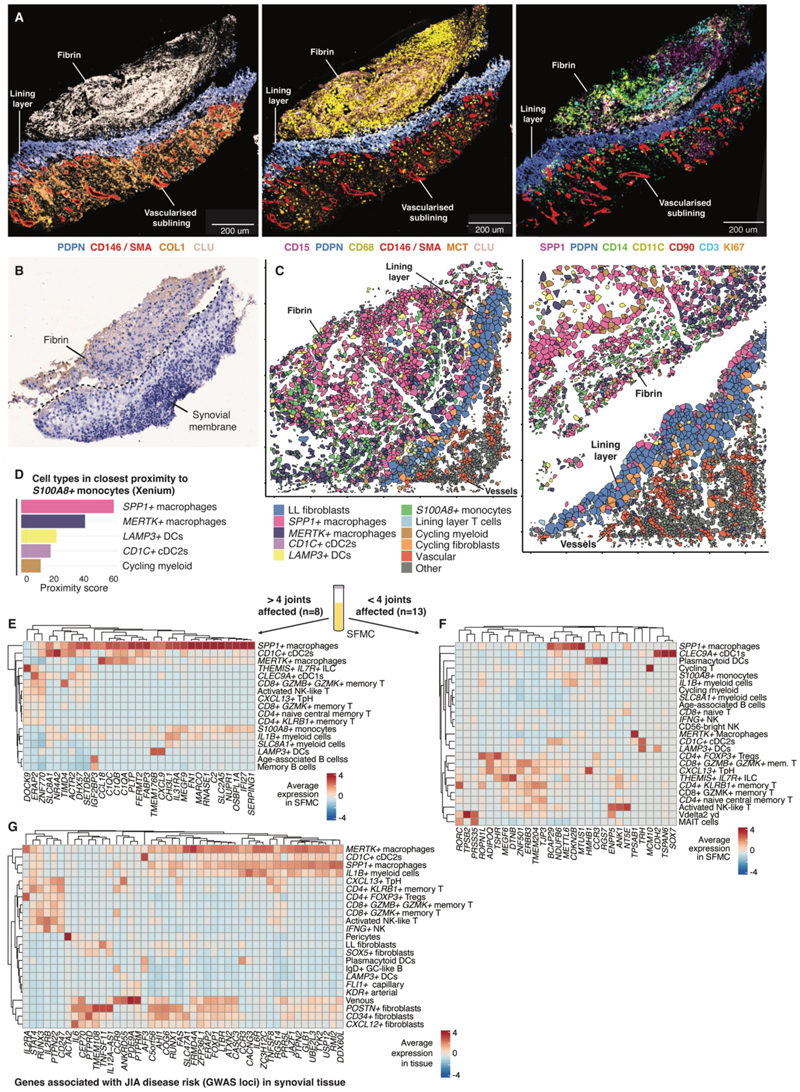
Genes associated with JIA disease risk and severity are expressed most highly in myeloid cell types present in the lining layer niche and fibrin deposits. (**A**) Representative multiplexed immunofluorescence image of *CLU+* fibrin deposit alongside *PDPN+* synovial tissue lining layer. Target proteins and color annotated under each image. Scale bars, 200 µm. (**B**) CD68+ immunohistochemical stain of synovial biopsy tissue showing the fibrin deposit alongside synovial tissue. (**C**) Spatial transcriptomic plots of regions containing *S100A8+* monocytes, showing fibrin fragments containing *SPP1+* macrophages, adjacent to the lining layer of a synovial tissue fragment. (**D**) Proximity analysis of cell types closest to *S100A8+* monocytes from spatial transcriptomics, *n* = 8. (**E and F**) Heatmap showing average expression of differentially expressed genes (>2 fold change, unadjusted *P* < 0.05) from SFMC obtained in early disease, which were associated with a subsequent disease course of more severe and extensive disease (> 4 joints, extended oligoarticular JIA, *n* = 8) (E) or more limited joint involvement (< 4 joints, persistent oligoarticular JIA, *n* = 13) (F). Gene set from reference: (28). Heatmap shows the 5 highest expressing cell types per gene. Color scale, average expression of genes in SFMC from our scRNA-seq data, *n* = 9. (**G**) Heatmap showing average expression of JIA genome-wide association study (GWAS) susceptibility loci (30, 50) in synovial tissue scRNA-seq from participants with JIA, *n* = 10. Hierarchical clustering analysis identifies groups of genes enriched across cell lineages and particular cell states. Top 3 cell types shown with the highest expression per gene. Color scale, average expression.

Multiplexed immunofluorescence revealed an additional niche of fibrin-associated cells distinguishable by proximity analysis and confirmed by cross-comparison with histological assessment (Fig. 6, A and B, fig. S7, D and E). Fibrin is an insoluble protein that aggregates within the synovial cavity following extravascular activation of the clotting cascade and can be gradually absorbed into the synovial membrane (24). Fibrin deposits were identified on multiplexed immunofluorescence images by the absence of vessels or lining layer, a distinct texture from synovium, low expression of collagens, and high expression of the glycoprotein clusterin (*CLU*), an extracellular chaperone that promotes aggregate formation and is abundant in clots (25, 26) (Fig. 5B, Fig. 6A, fig. S7, D and E). Proximity analysis showed a grouping of neutrophils and myeloid cells within fibrin deposits (group 3 in Fig. 5C; Fig. 6A). We observed in our spatial transcriptomic data that lining layer fibroblasts upregulated prothrombotic fibronectin (*FN1*), the *CLU* gene and pathways associated with platelet degranulation, suggesting involvement in fibrin deposition (Fig. 3E, fig. S3A).

Circulating S100A8/9 protein, produced by *S100A8+* monocytes, as well as by neutrophils, are one of the few available biomarkers predictive of flare in JIA, so we sought to localize these cells in synovial tissue (27). *S100A8*+ monocytes represented the only immune cell type in the lining layer niche which were not tissue-enriched, instead being uniquely more abundant in PBMC samples (Fig. 3A, Fig. 4, B and C). Proximity analysis of spatial transcriptomic data revealed that *S100A8+* monocytes were most likely to be located close to *SPP1+* macrophages in the synovial lining layer, as well as in tissue fragments close to the lining layer that resembled fibrin deposits, where *SPP1+* macrophages form aggregates (Fig. 6, C and D, fig. S7F). Other myeloid cells found in the lining layer niche, including *MERTK+* macrophages, *LAMP3+* DC and *CD1C+* cDC2, were also found in closest proximity to *S100A8+* monocytes, and their presence in fibrin deposits could be confirmed on multiplexed immunofluorescence images (Fig. 6A). This suggests that *S100A*8+ monocytes migrate into the tissue before infiltrating fibrin deposits alongside other tissue-resident myeloid cells.

### Genes implicated in susceptibility of JIA and risk of progression to severe disease are enriched in *SPP1+ MERTK-CD206-* macrophages

We next examined the expression of genes which we previously reported to be differentially expressed in the SFMC of children whose arthritis, though limited to ≤ 4 joints at time of diagnosis, extended to multiple other joints within 6 months (extended oligoarticular JIA, *n* = 8), compared with children whose arthritis remained limited (persistent oligoarticular JIA, *n* = 13) in our SFMC data (Fig. 6, E and F)(28). We found the genes that were associated with a severe arthritis trajectory were enriched in *SPP1+* macrophages that were *MERTK-CD206-* but expressed *TREM2* and pro-inflammatory *CXCL10* and *S100A8* (Fig. 6E, fig. S3A, fig. S7G). Several of these genes related to phagocytosis, including multiple *C1Q* genes, *MARCO* and *TIMD4* (Fig. 6E). Additionally, from this analysis, we noted that *LAMP3+* DC, typically considered tolerogenic, were associated with high expression of Th1-promoting T cell chemoattractant *CXCL9* (29). In contrast, genes upregulated from children whose arthritis remained limited to a few joints were enriched in T cell lineages, including unconventional T cells (MAIT cells and Vd2+ gamma-delta T cells) and CD8+/CD4+ memory cells (T peripheral helper, *GZMK+GZMB+* T cells), as well as in *CLEC9A+* cDC1 and pDC (Fig. 6F).

Mapping expression of susceptibility loci from two large genome-wide association studies of JIA onto our scRNA-seq synovial tissue dataset highlighted enrichment in similar lineages, with the addition of a stromal cell group (Fig. 6G) (30, 31). A large group of JIA risk genes (*TYK2, FAS*) were strongly expressed by myeloid and stromal cells, with enrichment in *MERTK+* and *SPP1+* macrophages. Another group of genes were specifically highly expressed by stromal cells (*IL6, TNFSF11*) with high expression in venous cells and *POSTN+* fibroblasts; and another by T cell subsets (*IL2RB, PTPN22, RUNX3*) with enrichment in *CXCL13+* T peripheral helper cells and activated NK-like T cells. Given the tissue-enrichment of these cell types, this highlights the importance of tissue resident cells as key drivers of disease pathology in JIA.

### JIA synovial tissue biopsies are more homogeneous than those from adults with RA, irrespective of clinical classification

**Figure 7:**
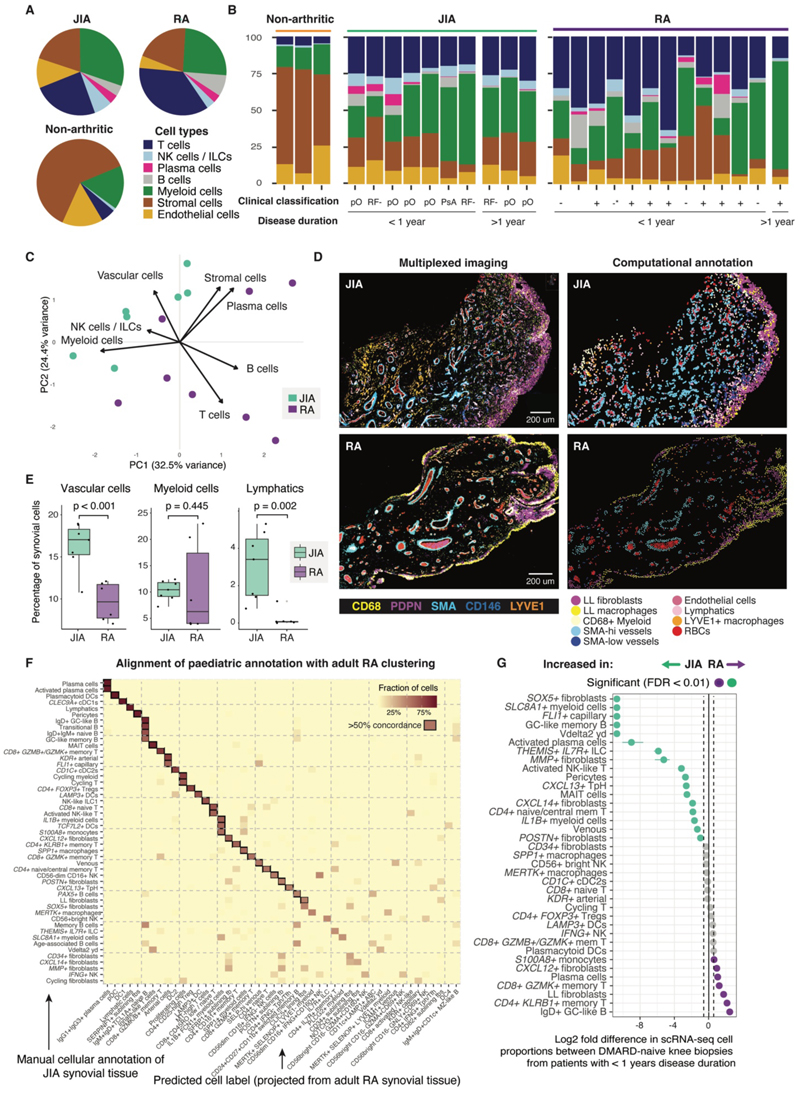
Distinct and shared features of the inflamed joint in children with JIA compared with adults with RA. (**A**) Comparative analysis of cell type proportions in synovial tissue of knee joints from adults with RA (*n* = 12, (13)), children with JIA (*n* = 7), and non-arthritic adults (*n* = 3, (8)) (enumerated from scRNA-seq). In arthritic cohorts, synovial tissue samples were derived from biopsies of DMARD-naïve individuals with a disease duration under one year. (**B**) Bar chart showing composition of individual synovial tissue samples by main cell types of all knee biopsies from children with JIA (*n* = 10), adults with RA (*n* = 13) and non-arthritic individuals (*n* = 3) from scRNA-seq data, irrespective of disease duration at time of biopsy; bars represent individual participant samples; disease duration indicated underneath. For JIA samples, ILAR clinical classification is indicated below: pO = persistent oligoarticular; RF- = rheumatoid factor negative polyarticular; PsA = psoriatic arthritis. For RA samples, American College of Rheumatology (ACR) / European Alliance of Associations for Rheumatology (EULAR) 2010 serology classification is indicated below: high positive rheumatoid factor (RF) / anti-citrullinated protein antibody (ACPA) (+); negative RF & ACPA (-); low ACPA (-*); RF unknown (). (**C**) PCA and biplot of synovial tissue cell type composition from children with JIA and adults with RA. Arrow length represents magnitude of impact on data variance; arrow direction represents correlation with principal components. Dots represent individual samples. (**D**) Representative examples of multiplexed immunofluorescence (left) and matched computational annotation (right) of synovial tissue from JIA synovial tissue (top) and RA synovial tissue (bottom). Scale bar, 200 µm. (**E**) Quantification of proportions of cell types found in multiplexed immunofluorescence images of JIA (*n* = 7), and RA synovial tissue samples (*n* = 6). Dots, individual participant samples. Box plots show median, IQR and highest/lowest value within 1.5 * IQR; Unadjusted P as shown; statistical tests used: vascular cells, t test; myeloid cells and lymphatics, Wilcoxon rank sum. (**F**) Plot showing concordance between JIA cluster annotation and predicted labels following label transfer from full RA (*n* = 69) to JIA (*n* = 10) synovial tissue scRNA-seq datasets. Color scale indicates the fraction of cells in JIA subclusters labelled with RA cluster annotation (label overlap). Black square outline indicates >50% concordance. Clusters with >15% concordance visualized. Fbs, fibroblasts. (**G**) Comparative analysis of proportional differences in inflamed RA adult and pediatric JIA joints in synovial tissue scRNA-seq. Samples as in (A), clusters labelled using label transfer from JIA to RA datasets. Significantly enriched cell types in JIA samples (green) and adult RA samples (purple) are shown (FDR < 0.01 (Fisher’s exact test and 10,000 permutations), > 0.58 log2 fold change). Non-significant findings shown in grey. Cell states shown that composed >0.5% of cells in synovial tissue.

To understand the distinctions between the inflamed tissue microenvironment in children with JIA compared with adults with RA, we compared our scRNA-seq data to a well-characterized adult synovial tissue dataset (13). We sought to limit confounders by comparing scRNA-seq data of synovial tissue from knee biopsies of DMARD-naïve individuals with a disease duration under one year in both cohorts (Fig. 7A, table S10). In a direct comparison between arthritic states, children with JIA had more myeloid cells, NK/ILC and vascular cells on average, whereas adults with RA had more T and B cells (Fig. 7, A to C). Myeloid cells were the most abundant cell type detected in 7 of 10 tissue samples from children with JIA, comprising 34.6% ± 14.4% of cells (Fig. 7, A and B). Furthermore, comparison of tissue composition by PCA showed separation between JIA and adult RA tissue samples (Fig. 7C). Samples from children with JIA were more homogeneous than samples from adults with RA, despite the inclusion of three different ILAR JIA clinical subtypes in this analysis (Fig. 7, B and C). We also performed a comparative analysis of multiplexed immunofluorescence images of RA and JIA synovium, using the previously described workflow (fig. S8A, table S11). Comparing cell numbers from annotated multiplexed immunofluorescence images between these age groups confirmed increased vascularity of JIA tissue compared with adult RA, and a significantly increased proportion of lymphatic vessel cells in JIA synovial tissue (*P* = 0.002, Fig. 7, D and E).

Transferring cell labels between pediatric JIA and adult RA synovial tissue scRNA-seq datasets enabled the identification of corresponding cell types between cohorts based on shared transcriptomic features (fig. S8B). Several pathogenic populations identified in adult RA (32) were also present and transcriptionally similar in children with JIA, including B cell-augmenting *CXCL13+* T peripheral helper cells and IgG1+IgG3+ plasma cells (Fig. 7F). In accordance with analysis of multiplexed immunofluorescence images, comparing cell type abundance in adult RA and JIA datasets revealed that capillary (*FLI1+/SPARC+*) and venous cells were more abundant in JIA synovial tissue in whichever direction label transfer was applied (FDR < 0.01; Fig. 7G, fig. S8C). Lymphatic cells were confirmed to be more abundant in the JIA samples (fig. S8C, fig. S9). Among ILC/NK cell types, ILC were particularly enriched in JIA samples (Fig. 7G, fig. S8C). Of the cell states that were more abundant in younger children in the JIA cohort, activated plasma cells and *POSTN+* fibroblasts were also enriched in JIA samples compared with RA samples (Fig. 7G, fig. S5A), raising the possibility that their enrichment reflects age-related differences, rather than disease distinctions.

### TGF-β-driven progenitor-like fibroblast states are uniquely enriched in JIA synovium

**Figure 8:**
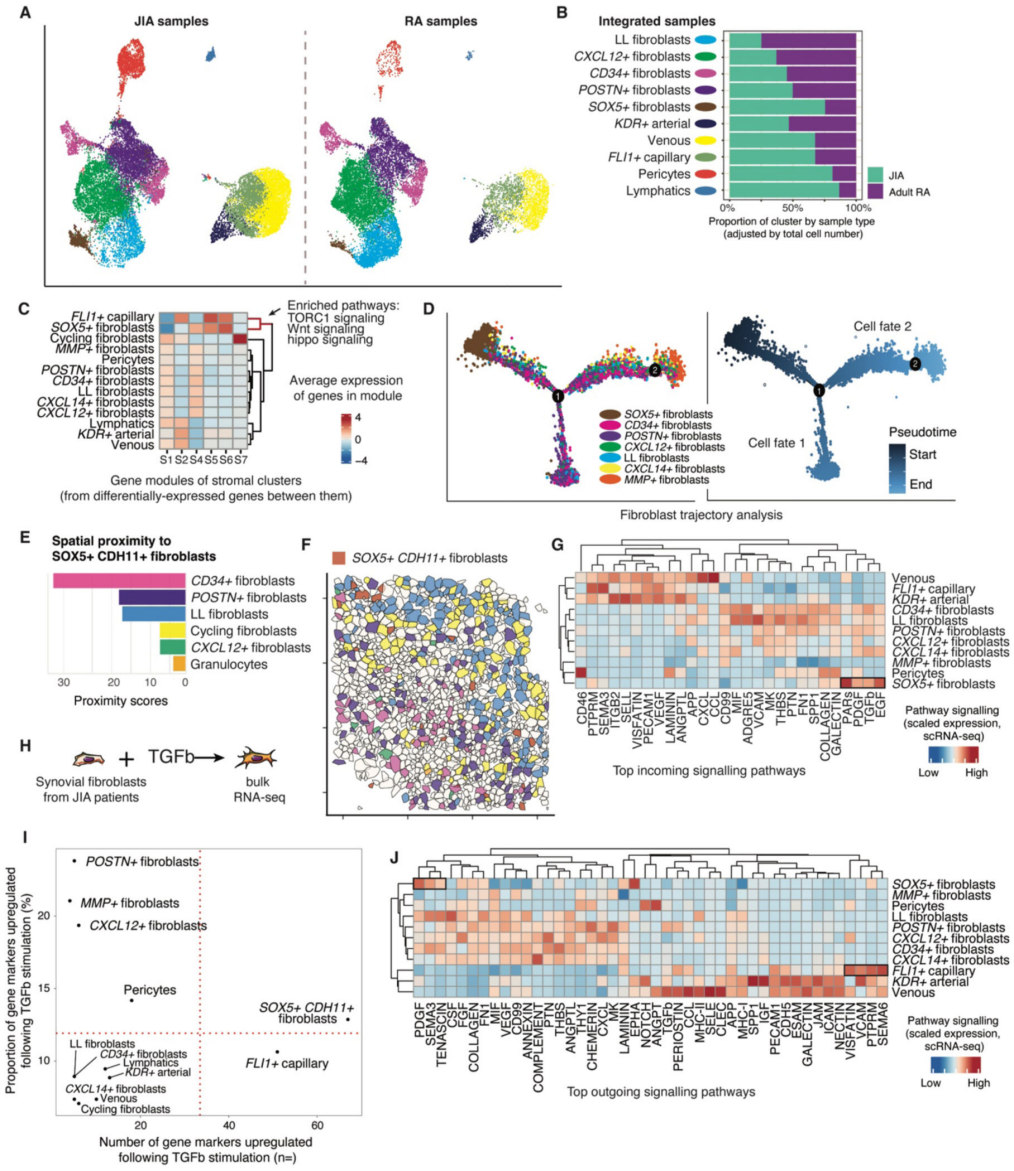
A progenitor-like fibroblast cluster is enriched in pediatric tissue samples. (**A**) Re-annotation of stromal cell types following integration and clustering of JIA (*n* = 7) and RA (*n* = 12) synovial tissue samples from DMARD-naive participants with disease duration < 1 year. (**B**) Bar plot showing proportions of re-annotated cell clusters by disease. (**C**) Analysis of modules of differentially expressed genes between stromal cell clusters in JIA synovial tissue, *n* = 10. Data presented as heatmap of average gene expression in the module with hierarchical clustering (full analysis in fig. S10D). Color scale, average expression of genes in module. (**D**) Pseudotime trajectory analysis of fibroblast populations from JIA synovial tissue scRNA-seq, *n* = 10. Left panel, cells colored by subcluster annotation. Right panel, cells colored by pseudotime trajectory. (**E**) Proximity analysis of cell types closest to *SOX5+CDH11+* fibroblasts in spatial transcriptomics, *n* = 8. Proximity scores indicate likelihood of cell types being the closest nearest neighbor. (**F**) Representative example of *SOX5+CDH11+* fibroblast distribution in spatial transcriptomic data. Polygon color, cell types indicated in (E). (**G**) Predicted incoming signaling pathways from ligand-receptor analysis of stromal cells (identified from scRNA-seq), synovial tissue *n* = 10. Top pathways per stromal cluster shown (tile color, communication probability of ligand-receptor interactions summarized in signaling pathways; columns, signaling pathways, unadjusted *P* < 0.05). (**H**) Schematic of experimental set-up for bulk RNA sequencing of TGF-β-stimulated (10 ng/mL) JIA synovial fibroblasts (from *n* = 3 children, 1 independent experiment). (**I**) Number (x axis) and proportion (y axis) of significantly upregulated genes (adjusted *P* < 0.05) following TGF-β stimulation that overlap with each stromal cell state’s marker genes (from scRNA-seq). Red dotted lines, half maximum value. (**J**) Top predicted outgoing signaling pathways from ligand-receptor analysis of stromal cells from JIA synovium, synovial tissue *n* = 10. Tile color, communication probability of ligand-receptor interactions summarized in signaling pathways; columns, signaling pathways (unadjusted *P* < 0.05).

*SOX5+* fibroblasts were strongly enriched in synovial tissue from children with JIA compared with adults with RA (FDR < 0.01) (Fig. 7G). The transcriptomic profile overlapped with both *CLIC5+* lining layer fibroblasts and *CD34+* sublining fibroblasts in the adult RA scRNA-seq data (Fig. 7F). Abundance of this cell type was not correlated with age (fig. S10A). To ensure the relative enrichment of *SOX5+* fibroblasts was not a technical artefact of cell label projection, we integrated JIA and RA stromal populations and re-annotated the integrated cell states, which confirmed the finding (Fig. 8, A and B, fig. S10, B and C). Differential gene expression analysis of all stromal cells from JIA synovial tissue revealed two gene modules (S5 & S6 modules) upregulated in both *SOX5+* fibroblasts and *FLI1+* capillary cells (Fig. 8C), another cell type enriched in JIA compared with adult tissue (Fig. 8, A and B, Fig. 7G). The genes in the S6 module were associated with WNT and HIPPO signaling, critical pathways linked to joint development/homeostasis and organ growth respectively (Fig. 8C, fig. S10D)(33, 34). *SOX5+* fibroblasts did not highly express the universal fibroblast progenitor marker *PI16* identified in adult datasets (13) (fig. S3A). Nonetheless, pseudotime trajectory analysis suggested that *SOX5+* fibroblasts were the origin of emergent fibroblasts states terminating in either lining layer/*MMP+* fibroblasts or collagen-expressing sublining fibroblast states (*CD34+ / POSTN+*; Fig. 8D). Consistent with these observations, spatial transcriptomic analysis showed that *SOX5+* fibroblasts were found in closest proximity to *CD34+* fibroblasts, *POSTN+* fibroblasts and lining layer fibroblasts, the final differentiation states suggested by trajectory analysis (Fig. 8, E and F).

Signaling from epidermal growth factor (EGF), platelet-derived growth factor (PDGF) and TGF-β pathways were upregulated in *SOX5+* fibroblasts (Fig. 8G). To verify the role of TGF-β signaling in influencing this cell state, we performed ex vivo stimulation of synovial fibroblasts cultured from synovial tissue biopsies from children with JIA using TGF-β, followed by bulk RNA sequencing (Fig. 8H, table S12). Synovial fibroblasts stimulated with TGF-β showed upregulation of genes that strongly overlapped with *SOX5+* fibroblast and *FLI1+* capillary markers, suggesting TGF-β plays a role in promoting the emergence of these subsets in JIA (Fig. 8I). As a proportion, there was also a large overlap with *POSTN+* fibroblast markers following TGF-β stimulation (Fig. 8I), suggesting a strong influence of this cytokine on this disease-associated population (Fig. 6G, fig. S4D). The strongest outgoing signals from *SOX5+* fibroblasts came from the pro-angiogenic mitogenic PDGF pathways, suggesting they may promote the vascularization of synovial tissue (Fig. 8J). Distinctions in anatomical structures, cellular proportions and stromal cell states between children with JIA and adults with RA further emphasizes the need for pediatric-focused tissue research.

## Discussion

In this study, we present a comprehensive single-cell and spatial multi-omic tissue atlas of the inflamed synovium in JIA. We elucidated the cellular heterogeneity of the tissue landscape, including the identification of rare and previously under-studied cell types, and distinguish immune cell phenotypes in synovial tissue from those in synovial fluid and blood. Our analysis identified age-correlated differences in the composition of myeloid and plasma cells within synovial tissue, highlighting their spatial organization within distinct tissue niches and their associated cellular signaling networks. We demonstrated an enrichment of JIA disease risk and severity genes in *SPP1+* tissue macrophages and show that cells upregulating disease-associated biomarkers co-localize with *SPP1+* macrophages in the lining layer. Additionally, these cell types are found within fibrin deposits in the synovial cavity, suggesting a potential role for fibrin processing in disease pathogenesis.

A critical barrier to implementing precision medicine strategies in JIA is our limited understanding of the cellular and molecular heterogeneity of the disease at the site of inflammation, which is not reflected in current classifications of JIA (7). In our data, tissue composition at diagnosis diverged most clearly in the abundance of plasma and myeloid cells and could not be distinguished by histological synovitis scores. Our results are consistent with previous studies that have profiled JIA PBMC/SFMC samples and tissue biopsies with bulk RNA sequencing, which found the main drivers of heterogeneity amongst individuals with JIA were related to the upregulation of B cell, plasma cell/immunoglobulin or myeloid cell signatures, irrespective of ILAR subtype (35, 36). Our scRNA-seq analysis of synovial tissue also aligns with gene profiles identified previously in JIA PBMC (36), where increased plasma cells were associated with a younger age of JIA onset and myeloid cells were more abundant in older children. Such age-specific cellular correlations could have important implications for the strategic application of targeted therapeutics. BAFF inhibitor treatments, such as belimumab, have been successfully employed in the treatment of other autoimmune diseases, including systemic lupus erythematosus and rheumatoid arthritis (RA) to reduce survival of autoreactive B cells. Given the enrichment of BAFF-related signaling in the plasma cell niche, BAFF inhibition may offer a pharmacological route for perturbing this niche (19).

In human adult and murine studies of synovium, macrophages highly expressing *TREM2* and *MERTK* are ascribed an immunoregulatory function in suppressing joint inflammation, with production of inflammation-resolving lipid mediators (6, 10). In contrast, *SPP1+MERTK-CD206-*myeloid cells have been characterized as inflammatory infiltrating cells in RA, with *SPP1+* macrophages absent in healthy tissue (10, 37). Strikingly, we find genes associated with arthritis severity (progression to extended oligoarticular disease) and risk of JIA to be strongly enriched in *SPP1+* macrophages. This cell type appears to acquire a pro-inflammatory program, expressing *CXCL10* and alarmins (*S100A8/S100A12*), but also genes typically associated with tissue residency (*TREM2, TIMD4*) (37). *SPP1+* macrophages localized to the outermost lining layer of the synovium in those with JIA, raising the question of whether a functional transition occurs in the barrier-like *TREM2+* macrophages. Polymorphic variants in the *SPP1*/osteopontin gene have been directly associated with a more severe disease course in JIA (38), suggesting this pleiomorphic glycoprotein has an active role in disease, rather than being simply a marker of a pathogenic cell state.

In JIA, the S100 calcium-binding alarmin proteins (*S100A8, S100A9*, and *S100A12*) have been identified as biomarkers for risk of flare upon withdrawal of methotrexate therapy and for identifying individuals less likely to respond to T cell targeting agents (27, 39–41). In our study, we show that cell types expressing elevated alarmins (neutrophils, *SPP1+* macrophages and *S100A8+* monocytes) are present in fibrin deposits in the synovial cavity. Fibrin deposition is an established feature of the inflamed synovium in JIA and impaired fibrin degradation from reduced fibrinolysis has been reported previously (42–44). Gene signatures related to coagulation and platelet function have been prominently detected in PBMC of patients with JIA and an older onset age of disease, in addition to the myeloid signature previously described (36). Recent studies have shown increased fibrin deposits and its precursor, fibrinogen, at an early stage in children who progress to more severe disease (42, 45). These data correspond with our own findings that genes upregulated early in the disease course of individuals who progress to a more severe and polyarticular disease were enriched in cell types present within fibrin deposits. Animal models have demonstrated that fibrin aggregates may directly drive invasive changes in fibroblasts, promote cartilage degradation and elevate expression of destructive enzymes (46), which suggests fibrin formation may not simply be a correlate of heightened inflammation, but an active pathogenic driver of disease.

Identifying molecular correlates of specific synovial tissue states from blood and synovial fluid remains an important step for predicting therapeutic response in a non-invasive manner. However, it is not currently known if such ‘liquid biopsies’ from blood or synovial fluid do indeed reflect tissue pathology. In the current study, we clearly define pathology related to resident cell biology in tissue that is not captured by analysis of PBMC or SFMC, including key signaling pathways, as well as immune and stromal cell states. With this unique opportunity to compare matched samples across anatomical compartments, we highlight distinctions in cellular composition and expression of compartment-specific transcriptional programs, for example the abundance of plasma cells in tissue and increased type-I IFN signaling in SFMC. These findings are important for contextualizing prior studies of JIA, highlighting that extrapolation of findings from SFMC analysis requires caution, and demonstrates the importance of studying the diseased tissue directly.

A detailed understanding of tissue immunology during development, across the age-spectrum and throughout the life-course of arthritis, especially in children, remains a substantial knowledge gap. There is a paucity of high-dimensional datasets available that incorporate childhood and adolescence. Our analysis is limited by an inability to compare with healthy age-matched tissue and across anatomically distinct joints (including load bearing and non-load bearing joints), however we do identify features from the inflamed knee synovium in JIA that are distinct from adults with RA and are therefore specific to this disease context. Careful interpretation is needed given the seropositive status of many of the adult RA participants, which may reflect differing underlying disease mechanisms to the seronegative participants with JIA. Although transcriptional diversity in fibroblasts has been demonstrated across anatomically distinct joints (47), initial scRNA-seq studies in RA have not identified populations unique to specific joints (13). However, appropriately powered well-matched studies are yet to be performed in either children or adults. Ultimately, our data emphasizes the need for a sufficiently powered tissue-based molecular stratification of JIA to guide therapeutic approaches and clinical care pathways in the pediatric population. To overcome the power limitations of the present study, in future we aim to provide a larger pathology-led molecular classification of JIA synovitis using a multi-center, biopsy-driven approach (TRICIA consortium: https://tricia.network/). This study represents an important step in tissue-based analytics in pediatric rheumatology by providing a detailed characterization of the immune and stromal landscape of the inflamed synovium in children; to move towards treatment stratification that is informed by molecular pathology and improve long-term outcomes for children with JIA.

## Materials and Methods

### Study design

The objective of this study was to characterize the cellular and spatial organization of the synovial microenvironment in children with JIA. Cellular composition and spatial proximity was assessed to identify disease-specific niches and molecular pathways. 19 children and young people with active JIA, who were less than 17 years of age and naïve to DMARD treatment at time of biopsy, were participants in this study, recruited from two UK pediatric rheumatology centers (table S1). Minimally invasive ultrasound-guided synovial tissue biopsies, synovial fluid aspirations and peripheral blood sampling were performed during routine clinical care while participants were receiving a therapeutic joint steroid injection under general anesthetic. 18/19 biopsies were obtained from the knee, the most commonly affected joint in children with JIA (48). Sample size was selected to capture a representative sample of the synovial architecture in JIA, incorporating children of different ages with multiple disease subtypes. Samples were excluded from sequencing protocols if disaggregated tissue yielded less than 40,000 live cells or if synovium was not detected by histological assessment of the biopsied tissue. Procedures were performed following written consent by the responsible parent or guardians, with age-appropriate participant assent where possible. Ethical approval was granted by the East of England - Cambridgeshire and Hertfordshire Research Ethics Committee (IRAS 292585, REC 21/PR/0410). For comparison of cellular composition, scRNA-seq datasets of arthritic (*n* = 13) and non-arthritic (*n* = 3) adult synovial tissue knee biopsies were obtained (8, 13). Tissue samples for multiplexed imaging comparisons were provided by adults with RA in the Birmingham Early Arthritic Cohort (BEACON) study who were DMARD-naïve and obtained by ultrasound-guided needle biopsy (49) (table S11). The BEACON study (12/WM/0258) was approved by the West Midlands Black Country Research Ethics Committee. All participants gave written, informed consent.

### Statistical analysis

All statistical analyses were performed in RStudio v4.1 or higher. Differential cell abundance of cell types from scRNA-seq was performed using scProportion test, utilizing Fisher’s exact test and random permutations to generate a *P*-value. An FDR threshold of 0.01 and a log2 fold change threshold of 0.58 was used for significance. Correlation analyses were performed by Spearman’s rank or Pearson’s correlation coefficient, depending on normality of data as assessed by the Shapiro-Wilk test. Pseudobulked differential expression was calculated using Wald test with Benjamini-Hochberg correction to calculate adjusted *P*-value in DESeq2, where an adjusted *P*-value threshold of 0.01 was applied. Proximity analysis *P*-value was derived from the z-score using the cumulative distribution function of the normal distribution with Benjamini-Hochberg correction to determine adjusted *P*-value. Gene ontology (GO) terms were determined using gsfisher and filtered on a *P*-value of < 0.05, calculated using Fisher’s exact test. For proportion comparisons of multiplexed immunofluorescence images, the t-test or Wilcoxon test was used depending on normality of data as assessed by the Shapiro-Wilk test. Bulk RNA sequencing differential expression was calculated using Wald test with Benjamini-Hochberg correction to calculate adjusted *P*-value in DESeq2, applying an adjusted *P*-value threshold of 0.05. All individual level data are available in **data file S1** or in GEO repositories at: GSE278962, GSE278968, GSE278969.

## Supplementary Material

Supplementary Materials

## Data Availability

All data associated with this study are in the paper or supplementary materials. The full analysis code, including custom analysis functions, is accessible at: https://github.com/crissi-b/MAPJAG and has been archived on Zenodo (https://doi.org/10.5281/zenodo.15352478). All newly sequenced data has been deposited in GEO repositories (GSE278962, GSE278968, GSE278969). Processed scRNA-seq data is available to view using the MDV platform: https://mdv.molbiol.ox.ac.uk/projects/mdv_project/7536 (registration required to access).

## One sentence summary

An atlas of the cellular and molecular landscape of the inflamed synovium in juvenile idiopathic arthritis defines tissue niches associated with disease severity

## References

[1] McHugh J (2020). Global prevalence of JIA, JSLE and club foot. Nat Rev Rheumatol.

[2] Oliveira-Ramos F, Eusébio M, Martins FM, Mourão AF, Furtado C, Campanilho-Marques R, Cordeiro I, Ferreira J, Cerqueira M, Figueira R, Brito I (2016). Juvenile idiopathic arthritis in adulthood: fulfilment of classification criteria for adult rheumatic diseases, long-term outcomes and predictors of inactive disease, functional status and damage. RMD Open.

[3] Shoop-Worrall SJW, Verstappen SMM, Baildam E, Chieng A, Davidson J, Foster H, Ioannou Y, McErlane F, Wedderburn LR, Thomson W, Hyrich KL (2017). How common is clinically inactive disease in a prospective cohort of patients with juvenile idiopathic arthritis? The importance of definition. Ann Rheum Dis.

[4] Taxter A, Donaldson BC, Rigdon J, Harry O (2022). Association Between Patient-Reported Outcomes and Treatment Failure in Juvenile Idiopathic Arthritis. ACR Open Rheumatol.

[5] Croft AP, Campos J, Jansen K, Turner JD, Marshall J, Attar M, Savary L, Wehmeyer C, Naylor AJ, Kemble S, Begum J (2019). Distinct fibroblast subsets drive inflammation and damage in arthritis. Nature.

[6] Alivernini S, MacDonald L, Elmesmari A, Finlay S, Tolusso B, Gigante MR, Petricca L, Di Mario C, Bui L, Perniola S, Attar M (2020). Distinct synovial tissue macrophage subsets regulate inflammation and remission in rheumatoid arthritis. Nat Med.

[7] Wedderburn LR, Ramanan AV, Croft AP, Hyrich KL, Dick AD (2022). Towards molecular-pathology informed clinical trials in childhood arthritis to achieve precision medicine in juvenile idiopathic arthritis. Annals of the Rheumatic Diseases.

[8] Tang S, Yao L, Ruan J, Kang J, Cao Y, Nie X, Lan W, Zhu Z, Han W, Liu Y, Tian J (2024). Single-cell atlas of human infrapatellar fat pad and synovium implicates APOE signaling in osteoarthritis pathology. Sci Transl Med.

[9] Matsubara T, Spycher MA, Rüttner JR, Fehr K (1983). The localization of fibronectin in rheumatoid arthritis synovium by light and electron microscopic immunohistochemistry. Rheumatol Int.

[10] Kurowska-Stolarska M, Alivernini S (2022). Synovial tissue macrophages in joint homeostasis, rheumatoid arthritis and disease remission. Nat Rev Rheumatol.

[11] Gray JI, Caron DP, Wells SB, Guyer R, Szabo P, Rainbow D, Ergen C, Rybkina K, Bradley MC, Matsumoto R, Pethe K (2024). Human γδ T cells in diverse tissues exhibit site-specific maturation dynamics across the life span. Science Immunology.

[12] Kumar BV, Ma W, Miron M, Granot T, Guyer RS, Carpenter DJ, Senda T, Sun X, Ho S-H, Lerner H, Friedman AL (2017). Human Tissue-Resident Memory T Cells Are Defined by Core Transcriptional and Functional Signatures in Lymphoid and Mucosal Sites. Cell Reports.

[13] Zhang F, Jonsson AH, Nathan A, Millard N, Curtis M, Xiao Q, Gutierrez-Arcelus M, Apruzzese W, Watts GFM, Weisenfeld D, Nayar S (2023). Deconstruction of rheumatoid arthritis synovium defines inflammatory subtypes. Nature.

[14] Straeten F, Zhu J, Börsch A-L, Zhang B, Li K, Lu I-N, Gross C, Heming M, Li X, Rubin R, Ouyang Z (2022). Integrated single-cell transcriptomics of cerebrospinal fluid cells in treatment-naïve multiple sclerosis. J Neuroinflammation.

[15] Morgan D, Tergaonkar V (2022). Unraveling B cell trajectories at single cell resolution. Trends in Immunology.

[16] Buechler MB, Pradhan RN, Krishnamurty AT, Cox C, Calviello AK, Wang AW, Yang YA, Tam L, Caothien R, Roose-Girma M, Modrusan Z (2021). Cross-tissue organization of the fibroblast lineage. Nature.

[17] Mayr CH, Sengupta A, Asgharpour S, Ansari M, Pestoni JC, Ogar P, Angelidis I, Liontos A, Rodriguez-Castillo JA, Lang NJ, Strunz M (2024). Sfrp1 inhibits lung fibroblast invasion during transition to injury-induced myofibroblasts. European Respiratory Journal.

[18] Conway SJ, Izuhara K, Kudo Y, Litvin J, Markwald R, Ouyang G, Arron JR, Holweg CTJ, Kudo A (2014). The role of periostin in tissue remodeling across health and disease. Cell Mol Life Sci.

[19] Kaegi C, Steiner UC, Wuest B, Crowley C, Boyman O (2021). Systematic review of safety and efficacy of belimumab in treating immune-mediated disorders. Allergy.

[20] Wei K, Korsunsky I, Marshall JL, Gao A, Watts GFM, Major T, Croft AP, Watts J, Blazar PE, Lange JK, Thornhill TS (2020). Notch signaling drives synovial fibroblast identity and arthritis pathology. Nature.

[21] De Klerck B, Geboes L, Hatse S, Kelchtermans H, Meyvis Y, Vermeire K, Bridger G, Billiau A, Schols D, Matthys P (2005). Pro-inflammatory properties of stromal cell-derived factor-1 (CXCL12) in collagen-induced arthritis. Arthritis Res Ther.

[22] Krenn V, Morawietz L, Burmester G-R, Kinne RW, Mueller-Ladner U, Muller B, Haupl T (2006). Synovitis score: discrimination between chronic low-grade and high-grade synovitis. Histopathology.

[23] Charabati M, Zandee S, Fournier AP, Tastet O, Thai K, Zaminpeyma R, Lécuyer M-A, Bourbonnière L, Larouche S, Klement W, Grasmuck C (2023). MCAM+ brain endothelial cells contribute to neuroinflammation by recruiting pathogenic CD4+ T lymphocytes. Brain.

[24] Sánchez-Pernaute O, López-Armada MJ, Calvo E, Díez-Ortego I, Largo R, Egido J, Herrero-Beaumont G (2003). Fibrin generated in the synovial fluid activates intimal cells from their apical surface: a sequential morphological study in antigen-induced arthritis. Rheumatology.

[25] Talens S, Leebeek FWG, Veerhuis R, Rijken DC (2019). Decoration of Fibrin with Extracellular Chaperones. Thromb Haemost.

[26] Schwochau GB, Nath KA, Rosenberg ME (1998). Clusterin protects against oxidative stress in vitro through aggregative and nonaggregative properties. Kidney International.

[27] Duurland CL, Wedderburn LR (2014). Current Developments in the Use of Biomarkers for Juvenile Idiopathic Arthritis. Curr Rheumatol Rep.

[28] Hunter PJ, Nistala K, Jina N, Eddaoudi A, Thomson W, Hubank M, Wedderburn LR (2010). Biologic predictors of extension of oligoarticular juvenile idiopathic arthritis as determined from synovial fluid cellular composition and gene expression. Arthritis Rheum.

[29] Lebre MC, Burwell T, Vieira PL, Lora J, Coyle AJ, Kapsenberg ML, Clausen BE, De Jong EC (2005). Differential expression of inflammatory chemokines by Th1- and Th2-cell promoting dendritic cells: A role for different mature dendritic cell populations in attracting appropriate effector cells to peripheral sites of inflammation. Immunology & Cell Biology.

[30] Hinks A, Cobb J, Marion MC, Prahalad S, Sudman M, Bowes J, Martin P, Comeau ME, Sajuthi S, Andrews R, Brown M, Boston Children’s JIA Registry, British Society of Paediatric and Adolescent Rheumatology (BSPAR) Study Group, Childhood Arthritis Prospective Study (CAPS), Childhood Arthritis Response to Medication Study (CHARMS), German Society for Pediatric Rheumatology (GKJR), JIA Gene Expression Study, NIAMS JIA Genetic Registry, TREAT Study, United Kingdom Juvenile Idiopathic Arthritis Genetics Consortium (UKJIAGC) (2013). Dense genotyping of immune-related disease regions identifies 14 new susceptibility loci for juvenile idiopathic arthritis. Nat Genet.

[31] López-Isac E, Smith SL, Marion MC, Wood A, Sudman M, Yarwood A, Shi C, Gaddi VP, Martin P, Prahalad S, Eyre S (2021). Combined genetic analysis of juvenile idiopathic arthritis clinical subtypes identifies novel risk loci, target genes and key regulatory mechanisms. Ann Rheum Dis.

[32] Rao DA, Gurish MF, Marshall JL, Slowikowski K, Fonseka CY, Liu Y, Donlin LT, Henderson LA, Wei K, Mizoguchi F, Teslovich NC (2017). Pathologically expanded peripheral T helper cell subset drives B cells in rheumatoid arthritis. Nature.

[33] Guo X, Day TF, Jiang X, Garrett-Beal L, Topol L, Yang Y (2004). Wnt/β-catenin signaling is sufficient and necessary for synovial joint formation. Genes Dev.

[34] Zhong Z, Jiao Z, Yu FX (2024). The Hippo signaling pathway in development and regeneration. Cell Reports.

[35] Triaille C, Tilman G, Baert CA, Sokolova T, Loriot A, Nzeusseu-Toukap A, Meric de Bellefon L, Galant C, Boulanger C, Fonseca JE, Bouzin C (2024). Two Broad Categories Overlapping With Rheumatoid Arthritis Observed in Synovial Biopsies from Patients With Juvenile Idiopathic Arthritis. Arthritis & Rheumatology.

[36] Barnes MG, Grom AA, Thompson SD, Griffin TA, Luyrink LK, Colbert RA, Glass DN (2010). Biologic similarities based on age at onset in oligoarticular and polyarticular subtypes of juvenile idiopathic arthritis. Arthritis Rheum.

[37] Hanlon MM, Smith CM, Canavan M, Neto NGB, Song Q, Lewis MJ, O’Rourke AM, Tynan O, Barker BE, Gallagher P, Mullan R (2024). Loss of synovial tissue macrophage homeostasis precedes rheumatoid arthritis clinical onset. Sci Adv.

[38] Marciano R, Giacopelli F, Divizia MT, Gattorno M, Felici E, Pistorio A, Martini A, RavazzolGe R (2006). Ann Rheum Dis.

[39] Brunner HI, Schulert GS, Sproles A, Thornton S, Cornejo GV, Antón J, Cuttica R, Henrickson M, Foeldvari I, Kingsbury DJ, Askelson M, on behalf of the Pediatric Rheumatology Collaborative Study Group (PRCSG) and the Paediatric Rheumatology International Trials Organisation (PRINTO) (2024). S100 proteins as potential predictive biomarkers of abatacept response in polyarticular juvenile idiopathic arthritis. Arthritis Research & Therapy.

[40] Foell D, Wulffraat N, Wedderburn LR, Wittkowski H, Frosch M, Gerss J, Stanevicha V, Mihaylova D, Ferriani V, Tsakalidou FK, Foeldvari I (2010). Paediatric Rheumatology International Trials Organization (PRINTO), Methotrexate withdrawal at 6 vs 12 months in juvenile idiopathic arthritis in remission: a randomized clinical trial. JAMA.

[41] Hinze CH, Foell D, Johnson AL, Spalding SJ, Gottlieb BS, Morris PW, Kimura Y, Onel K, Li SC, Grom AA, Taylor J (2019). Serum S100A8/A9 and S100A12 Levels in Children With Polyarticular Forms of Juvenile Idiopathic Arthritis: Relationship to Maintenance of Clinically Inactive Disease During Anti-Tumor Necrosis Factor Therapy and Occurrence of Disease Flare After Discontinuation of Therapy. Arthritis Rheumatol.

[42] Costi S, Armiraglio E, Pregnolato F, Chighizola CB, Marino A, Randelli PS, Parafioriti A, Caporali R (2023). Diagnostic and prognostic role of synovial tissue analysis in juvenile idiopathic arthritis: a monocentric study. RMD Open.

[43] Mussoni L, Pintucci G, Romano G, Benedetti FD, Massa M, Martini A (1990). Decreased fibrinolytic activity in juvenile chronic arthritis. Annals of the Rheumatic Diseases.

[44] Wynne-Roberts CR, Anderson CH, Turano AM, Baron M (1978). Light- and electron-microscopic findings of juvenile rheumatoid arthritis synovium: Comparison with normal juvenile synovium. Seminars in Arthritis and Rheumatism.

[45] Finnegan S, Robson J, Scaife C, McAllister C, Pennington SR, Gibson DS, Rooney ME (2014). Synovial membrane protein expression differs between juvenile idiopathic arthritis subtypes in early disease. Arthritis Res Ther.

[46] Hügle T, Nasi S, Ehirchiou D, Omoumi P, So A, Busso N (2022). Fibrin deposition associates with cartilage degeneration in arthritis. eBioMedicine.

[47] Frank-Bertoncelj M, Trenkmann M, Klein K, Karouzakis E, Rehrauer H, Bratus A, Kolling C, Armaka M, Filer A, Michel BA, Gay RE (2017). Epigenetically-driven anatomical diversity of synovial fibroblasts guides joint-specific fibroblast functions. Nat Commun.

[48] Heckert SL, Hissink-Muller PCE, van den Berg JM, Schonenberg-Meinema D, van Suijlekom-Smit LW, van Rossum MAJ, Koopman Y, ten Cate R, Brinkman DMC, Huizinga TWJ, Allaart CF (2023). Patterns of clinical joint inflammation in juvenile idiopathic arthritis. RMD Open.

[49] Choi IY, Karpus ON, Turner JD, Hardie D, Marshall JL, De Hair MJH, Maijer KI, Tak PP, Raza K, Hamann J, Buckley CD (2017). Stromal cell markers are differentially expressed in the synovial tissue of patients with early arthritis. PLoS ONE.

[50] Lopez MC, Tabatabaian F, Niebur H, Kellner E, Uzel G, Leiding J (2021). Common Variable Immunodeficiency in a young male uncovers Nuclear Factor kappab-1 (NFKB1) haploinsufficiency with variable phenotype in several relatives: The importance of pursuing a genetic diagnosis. Journal of Clinical Immunology.

[51] Krenn V, Morawietz L, Burmester G-R, Kinne RW, Mueller-Ladner U, Muller B, Haupl T (2006). Synovitis score: discrimination between chronic low-grade and high-grade 1322 synovitis. Histopathology.

