# Supplementary material for: Synovial tissue atlas in juvenile idiopathic arthritis reveals pathogenic niches associated with disease severity
Source: Sci Transl Med. Author manuscript; Available in PMC 2025 Jul 22. (PMC7617933; doi:10.1126/scitranslmed.adt6050)
Supplement: Supplementary Materials [file EMS206877-suppement-Supplementary_Materials.pdf]

List of Supplementary Materials

Supplemental Methods

Figures S1 to S10

Legends for tables S1 to S11

Other Supplementary Materials

Tables S1 to S12

MDAR

Data file S1

Supplementary Methods

Sample processing

Synovial tissue samples were dissected into 1-2 mm pieces, and 6-12 fragments were suspended in cryopreservation media (C2874, CryoStor CS10, Sigma-Aldrich). Remaining tissue was set aside for tissue culture and/or fixed in formalin (D8418, Sigma-Aldrich) overnight and then embedded in paraffin. Synovial fluid was first treated with hyaluronidase (H4272, Sigma-Aldrich) for 30 minutes at 37 °C. PBMC and SFMC were isolated by density centrifugation using Lymphoprep (85450, Stem Cell Technologies) with SepMate PBMC Isolation Tubes (85450, Stem Cell Technologies) and stored in freezing media (10% DMSO (D8418, Sigma-Aldrich) in non-heat inactivated fetal calf serum (Fetal Bovine Serum, FCS-SA, Labtech)) in aliquots of 5 million cells.

Histological assessment of synovial tissue

Synovial biopsy sections were formalin-fixed paraffin-embedded (FFPE) for histological analysis. Hematoxylin and eosin (H&E)-stained synovial biopsy sections were examined histologically for the degree of lining layer hyperplasia and the severity of inflammatory infiltrate using the Krenn synovitis score as previously described (*51*). Two pathologists provided independent scores and the mean of these scores was used for further analysis.

Preparation of samples for scRNA-seq

Tissue fragments were separated mechanically and enzymatically in digestion buffer (100 µg/mL Liberase TL (5401020001, Sigma-Aldrich) and 100 µg/mL DNase I (DN25, Sigma-Aldrich) in RPMI-1640 (21875034, Gibco) supplemented with 100 U/mL penicillin and 100 µg/mL streptomycin (15140122, Invitrogen)) in a 37 °C water bath for 30 minutes, with mechanical agitation at 15 minutes. Single cell suspensions from disaggregated synovial tissues were assessed for cell quantity and cell viability by trypan blue.

scRNA-seq library preparation and analysis

scRNA-seq libraries were prepared using Chromium Next GEM single-cell 5' kit (v2 Dual Chemistry, PN-1000265, 10x Genomics), Chromium Single Cell Human TCR/BCR Amplification Kit (PN-1000252 and PN-1000253, 10x Genomics) and TotalSeq-C Human Universal Cocktail (v1.0, 399905, BioLegend) and sequenced using the NovaSeq 6000 platform to a read depth of 50,000 reads for gene expression and 5,000 reads for VDJ sequencing/surface antigen detection. Alignment to the GRCh38 reference genome and generation of count matrices for each sample was competed using CellRanger v7.0.01 (10x Genomics).

scRNAseq analysis was completed using RStudio (v.4.1 for initial integration of data, v4.2 for downstream processing). SoupX (v1.6.2) was used to remove ambient mRNA contamination. Cells with a doublet score > 0.25 were excluded (scrublet v.0.2.3, python v.3.9). Data was analyzed in Seurat (v4.0.3, v5.0.0), where the following quality control metrics were used per cell: <200 or >7000 detected genes (nFeature_RNA), <12.5% of mitochondrial RNA counts, except innate lymphoid cells where <20% was applied, and for PBMC and SFMC samples,

<10,000 protein counts of surface antigens. Data was processed using the following Seurat functions: NormalizeData(), FindVariableFeatures(), RunPCA(), FindNeighbours(), RunUMAP(), FindClusters() and FindMarkers(). Samples were integrated using FindIntegrationAnchors() and IntegrateData() (reduction = “rpca”). Additional batch correction was performed where cellular subtypes were not well integrated using the Harmony package (v0.1). Different resolutions of clustering were considered and the expression, as well as the number, of variable features was inspected between clusters for annotation (**table S2 and S5**). AddModuleScore() was used to score cells by their aggregate expression of tissue-resident memory T cell markers.

Differential abundance analysis was performed using scProportionTest (v0.9). Differential expression of pseudobulked counts across specimen types/anatomical compartments was analyzed in DESeq2 (v1.40.2) (**table S3**), modules were generated using igraph (v1.4.2) and data was visualized using ComplexHeatmap (v2.14) or pheatmap (v1.0.12). Biological pathway analysis was performed using gsFisher (v0.2) (**table S4 and S6**). For interactome analyses, the CellChat package (v1.6.1) was used and adapted. Trajectory analysis was complete using monocle (v2.28.0). Average expression of disease-associated genes was performed with AggregateExpression() (Seurat v5). Cells were ordered using setOrderingFilter() and all marker genes for stromal cell clusters. Dimensions were reduced using reduceDimensions(max_components = 2, method = 'DDRTree') and ordered using orderCells(). For comparison of pediatric and adult datasets, cell identity labels were transferred from *(2)* using FindTransferAnchors() and TransferData(). Processed inflamed human synovium scRNAseq data was kindly provided by Prof. Wei and is publicly available from Synapse (https://doi.org/10.7303/syn52297840).

Spatial transcriptomics sample processing and data analysis

RNA and sample quality of FFPE tissue samples were evaluated using DV200 metrics and H&E staining. Slides were prepared, processed, imaged and underwent post-run staining according to Xenium (10x Genomics) protocols: “CG000580 Rev C”, “CG000582 Rev D”, “CG000584 Rev B” and “CG000613 Rev A”. For gene detection, slides hybridized with probes from the predesigned Xenium Human Multi-Tissue and Cancer Gene Expression and hMulti_v1 design (chemistry v1, 10x Genomics), consisting of 377 genes, was used (**table S7**).

Cells were segmented from raw decoded transcripts using baysor (v0.6.0) and a prior segmentation cell radius of 10μm. Segmented data was processed using the previously mentioned functions in Seurat (v4.3.0) (**table S8**)**.** Cells with <15 genes and <20 transcripts were removed. Cellular boundaries, proximity analysis and niche analysis was completed using: sfdct (v0.2.0), sf (v1.0-13), spatula (v0.9) and sp (v2.0-0). Samples were batch corrected across fields of view (FOVs) using harmony.

Multiplexed immunofluorescence imaging

One synovial tissue biopsy was analyzed using both spatial transcriptomics and multiplexed imaging, meaning samples provided an independent cohort for comparison. FFPE synovial tissue was sectioned at 4 μm, incubated for 1 hour at 60 ºC, deparaffinized and rehydrated according to the Leica Cell DIVE standardized protocol consisting of successive changes of Xylene (2x wash)

for 5 minutes each with gentle agitation, followed by 2x wash of 5 minutes each in 100%, 95%, 70% and 50% ethanol with gentle agitation, and two changes of phosphate-buffered saline (PBS) for 5 minutes each. Permeabilization was performed for 10 minutes in 1x PBS with 0.3% Triton X100, followed by a PBS wash of 5 minutes. Antigen retrieval was carried out using a pressure cooker, according to manufacturer's recommendations. Slides were then stained with DAPI and imaged for the first scan plan. Imaging was performed at 20X to acquire background autofluorescence and generate virtual H&E images. Each staining round consisted of 3 conjugated antibodies incubated at 4°C overnight or for an hour at room temperature (**table S9**). Manually conjugated antibodies were purchased in a BSA-Azide-free format and conjugated using antibody labelling kits (A20181, A20187, A20186, ThermoFisher). Between staining rounds, slides were bleached and re-stained with DAPI to assist in image registration and alignment. Anti-CD138 staining was unsuccessful and, therefore, plasma cells were subsequently visualized using confocal microscopy.

Confocal microscopy

FFPE paraffin embedded slides were deparaffinized and rehydrated, and antigen retrieval at Tris-EDTA pH9 performed for one hour at 95 °C (Dako Target Retrieval Solution, S2367, Agilent). Before antibody addition, slides were pre-treated with Image-iT FX Signal Enhancer (I36933, Invitrogen). Slides were incubated with the following antibodies overnight at 4 °C: anti-CD15, anti-CD138, anti-CD31 (**table S9**). Secondary antibodies were used to detect primary antibody bound to epitope. Autofluorescence quencher was used (Vector TrueVIEW Autofluorescence Quenching Kit, SP-8400, Vector Laboratories) and slides mounted with DAPI (VECTASHIELD Vibrance Anti-Fade Mounting Medium + DAPI, H-1800, Vector Laboratories) for visualization. Slides were imaged on a Zeiss LSM 880 confocal microscope and analysed in QuPath v0.5.0. Single-stain and isotype-matched controls were run to validate staining.

Computational analysis of multiplexed immunofluorescence imaging

Cell segmentation of Leica Cell DIVE images was performed in QuPath (v0.4.4). Cell detection was performed using the initial DAPI staining, with a background nucleus radius of 6 mm. Following segmentation to define cell boundaries, average fluorescence intensity was calculated across the nucleus and cytoplasm. Intensity matrices from each sample were read into R (v4.2.0), processed using sctransform and Seurat using the functions previously described. Proximity analysis was completed using korsunskylab/spatula (v0.9) and Matrix (1.6-0).

Fibroblast culture and cytokine stimulation

Up to 4 < 1 mm fragments of synovial biopsy tissue were cultured in RPMI-1640 media (31870025, Gibco™), which was supplemented with 10% non-heat inactivated fetal calf serum, 1% [100X] MEM Non-essential amino acids (M7145, Sigma-Aldrich), 1% [100 mM] Sodium Orthopyruvate (S8636, Sigma-Aldrich), 2 mM glutamine, 100 U/mL penicillin and 100 μg/mL streptomycin (G6784, Sigma-Aldrich). Fibroblasts were expanded until passage 3-6 for *in vitro* experiments. For TGF-b stimulation, cultured synovial fibroblasts (n = 3 biological replicates, all derived from female patients) were treated for 24 hours with 10 ng/mL recombinant human TGF-β (201-LB-005/CF, Bio-Techne) or DMSO (D8418, Sigma-Aldrich).

Bulk RNA sequencing sample preparation

RNA was extracted using ARCTURUS PicoPure RNA Isolation Kit (KIT0204, Thermo Fisher Scientific) according to the manufacturers' instructions. Library prep was completed using Novogene NGS RNA Library Prep Set and sequenced on a NovaSeq X Plus using Illumina with PE-150 strategy. Sequenced reads were aligned to the GRCh38 human genome using Bowtie2 (v2.4.4). PCR duplicates were removed using SMAtools (v1.15.1). Count matrices were generated using Subread (v2.0.1). Differential expression was completed using DESeq2 (v1.40.2) (**table S12**).

Sample attrition

Some samples were omitted from further analysis because they were found to be of insufficient quality or cell number for analysis. In the scRNA-seq analysis, this included 2 x tissue scRNA-seq samples. For comparisons between anatomical compartments (SFMC, PBMC and synovial tissue), only JIA samples with sufficient cell numbers in all 3 specimen types were included to ensure a fair comparison (n = 6). In 3 x H&E images, no lining layer was captured in the tissue fragments and this was therefore not scored for hyperplasia. Additionally, 2 x JIA multiplexed immunofluorescence images could not be analyzed as stain processing was unsuccessful.

Supplementary Figure 1

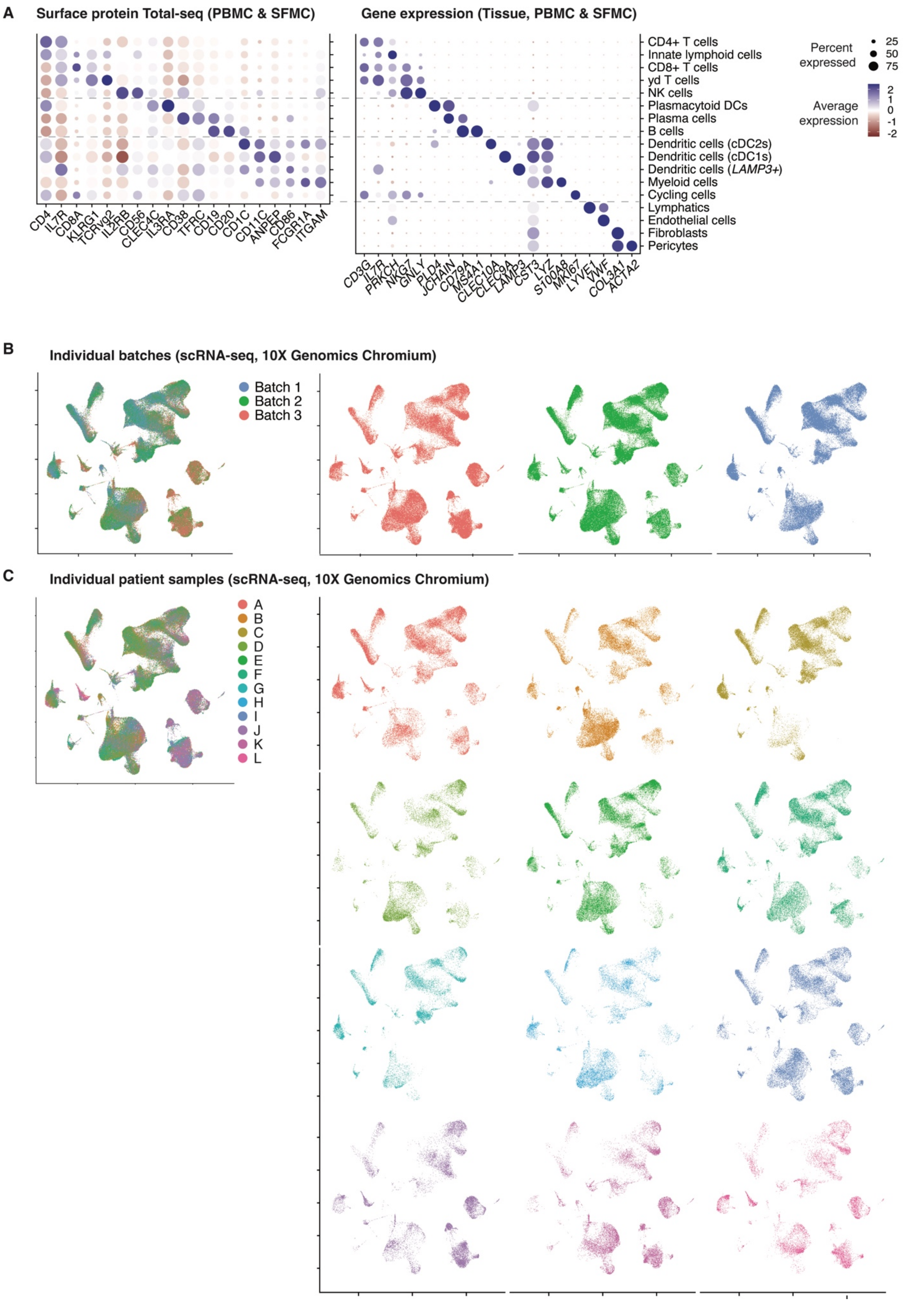

Figure S1: **Key transcriptomic and protein markers of the major cell types in the inflamed synovium of children with JIA.** (**A**) Average expression of cell surface antigens (CITE-seq: BioLegend TotalSeq) and genes to annotate main cell types found in JIA samples. (**B and C**) UMAP embeddings of integrated scRNA-seq from synovial tissue, PBMC and SFMC (n=12 participants) split by batch (B) and patient (C).

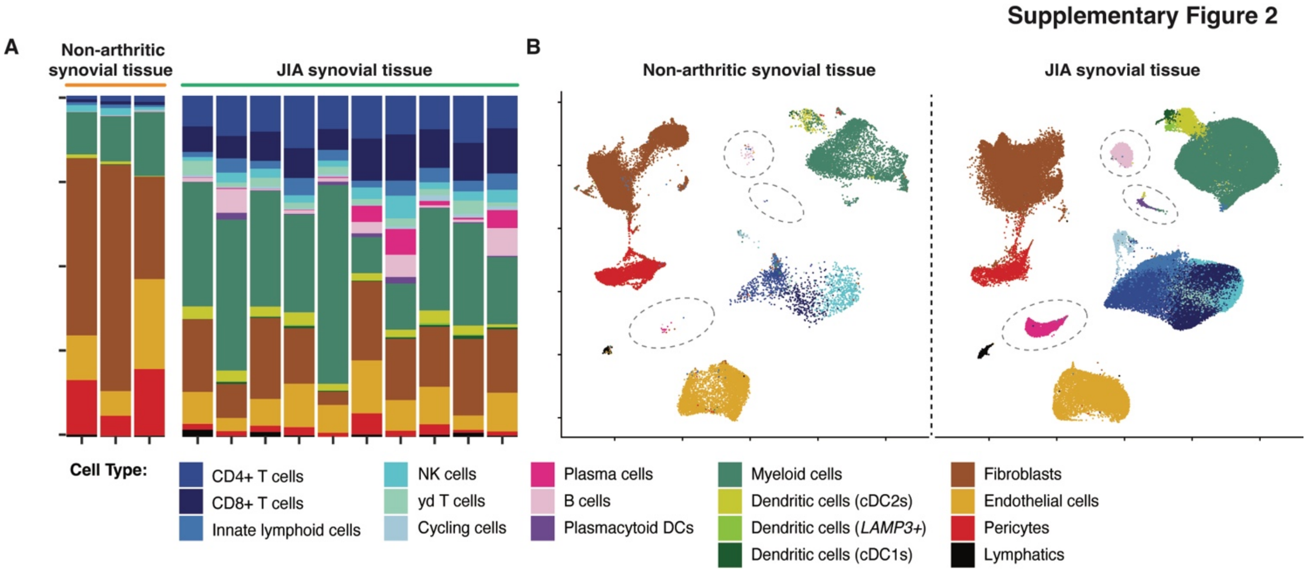

Figure S2: Comparative analysis of knee synovial tissue composition in JIA and non-arthritic adult samples. (**A**) Bar chart showing composition of individual synovial tissue samples by main cell types of all participants with JIA (*n* = 10, 106492 cells) and non-arthritic adults (*n* = 3, 34557 cells) from scRNA-seq data of knee biopsies. (**B**) Seurat-integrated UMAP embeddings of main cell types, split by disease status. Samples as per (A).

Supplementary Figure 3

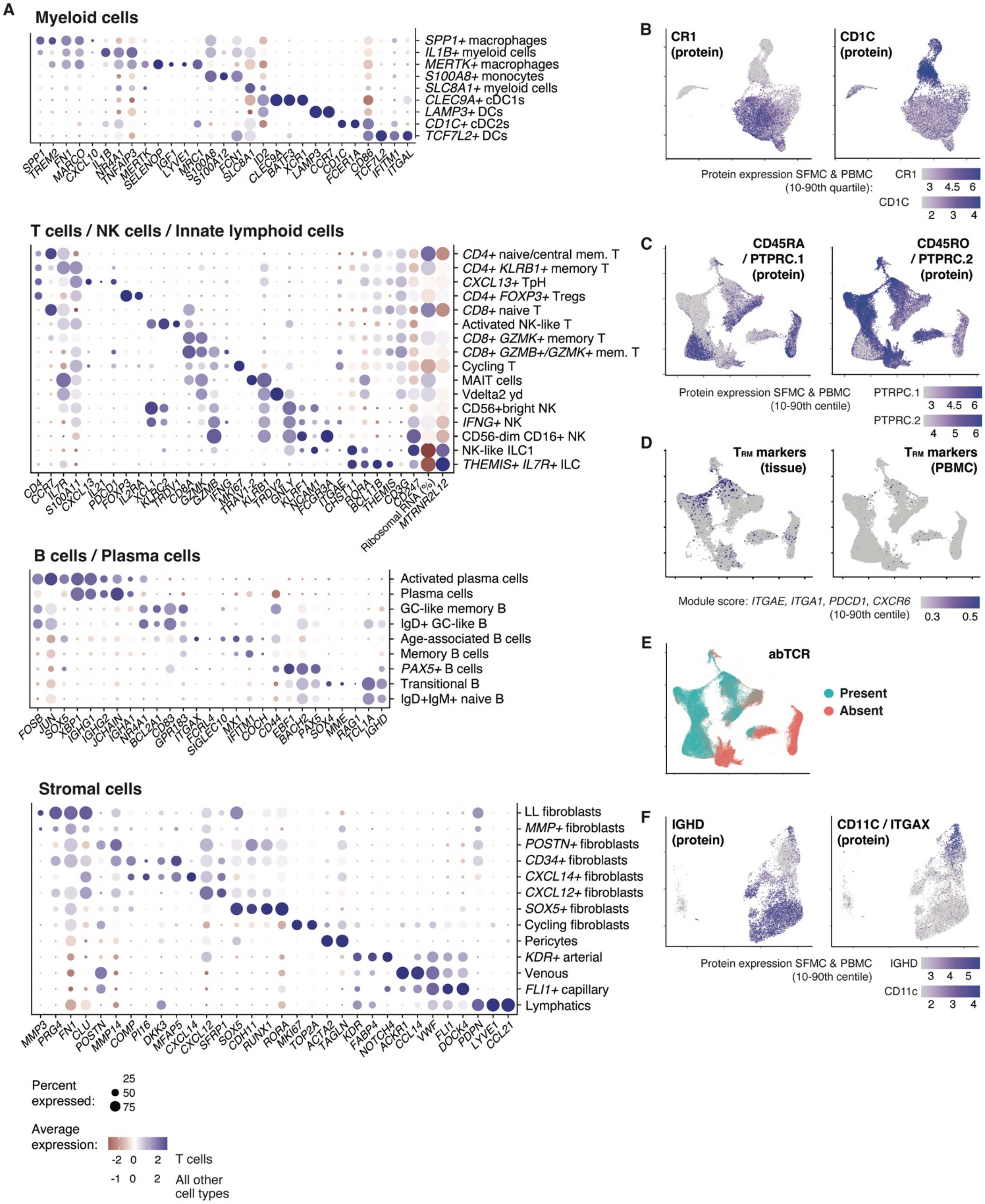

Figure S3: **Key transcriptomic and protein markers of the finely-clustered cell types in the tissue and cell samples from children with JIA.** (**A**) Average expression of gene markers identifying clusters from integrated scRNAseq analysis (synovial tissue, PBMC and SFMC, *n* = 12) at high cluster resolution across 4 cell lineages. Mem: memory. (**B**) Feature plot showing CITE-seq analysis of cell surface antigens in CR1+ monocytes/macrophages (left) and cDC2s/LAMP3+ DCs (right) in myeloid cells from PBMC and SFMC samples. (**C**) Feature plot showing cell surface antigen detection of naïve CD45RA+ T cells (left) and CD45RO+ memory T cells (right) in T cells/NK cells/innate lymphoid cells from PBMC and SFMC samples. (**D**) Feature plot of aggregate expression scores of resident memory (T_{RM}) markers in T cells/NK cells/innate lymphoid cells from tissue and PBMC samples. (**E**) Feature plot showing presence or absence of alpha-beta T cell receptors (abTCR) in PBMC + SFMC samples from cell surface antigen detection analysis to distinguish conventional T cells from unconventional T cells, ILC and NK cells. (**F**) Feature plot showing cell surface antigen detection of IgD+ naïve B cells (left) and ITGAX+ age-associated B cells (right) in B/plasma cells from PBMC and SFMC samples.

Supplementary Figure 4

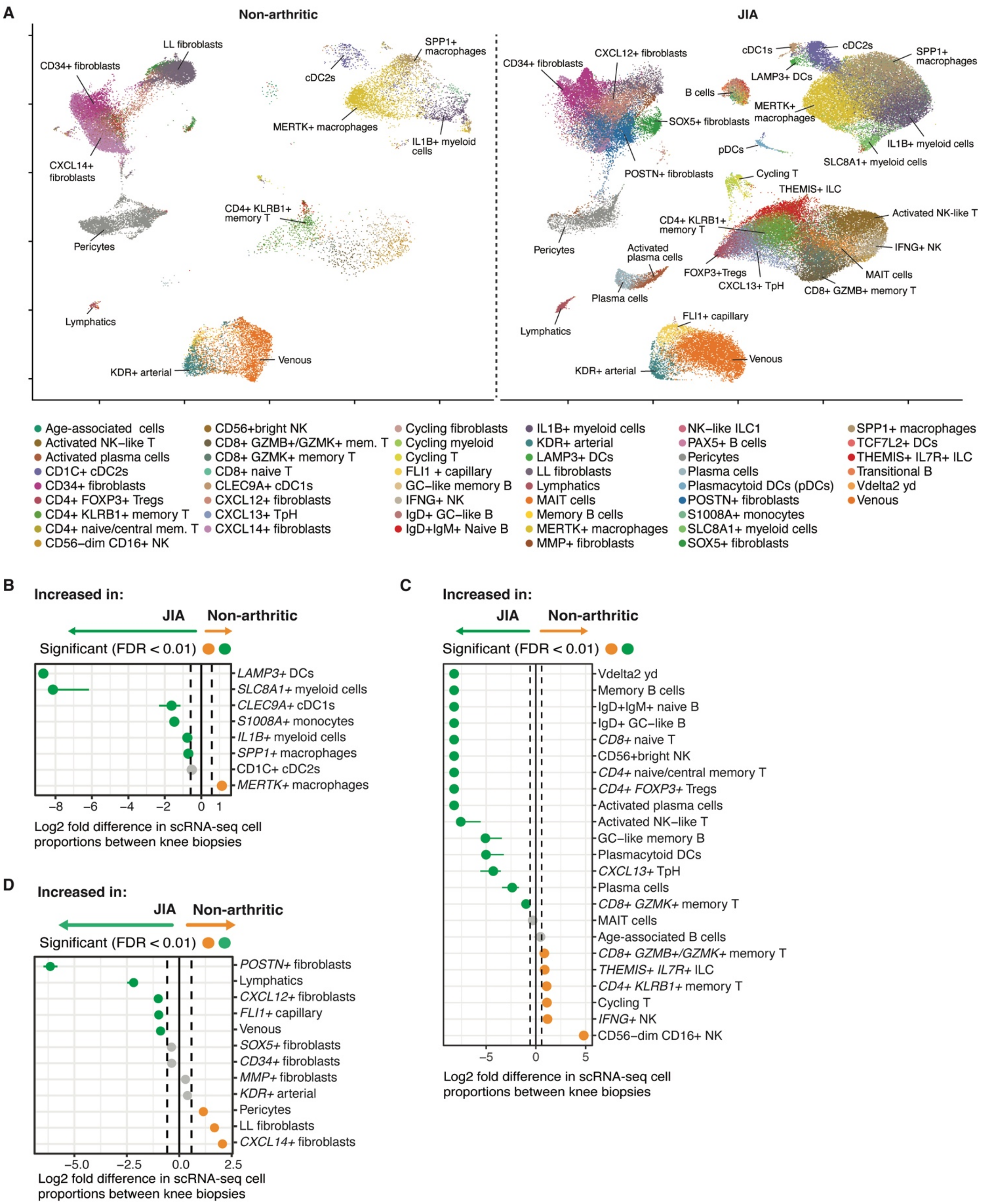

Figure S4: Comparative fine subcluster analysis of knee synovial tissue composition in JIA and non-arthritic adult samples. (**A**) Seurat-integrated UMAP embeddings of finer cell sub-clustering following label transfer split by disease status; JIA samples n = 10, n = 106492 cells; non-arthritic adult samples n = 3, n = 34557 cells. **(B-D**) Comparative analysis of proportional differences between non-arthritic adult and pediatric JIA knee biopsies across myeloid (**B**), lymphocyte (**C**) and stromal (**D**) populations, from synovial tissue scRNA-seq. Significantly enriched cell types in JIA samples (green) and non-arthritic adult samples (orange) are shown (FDR < 0.01 (Fisher's exact test and 1,000 permutations), > 0.58 log2 fold change). Non-significant changes shown in grey. Only clusters containing > 300 cells in tissue are visualized.

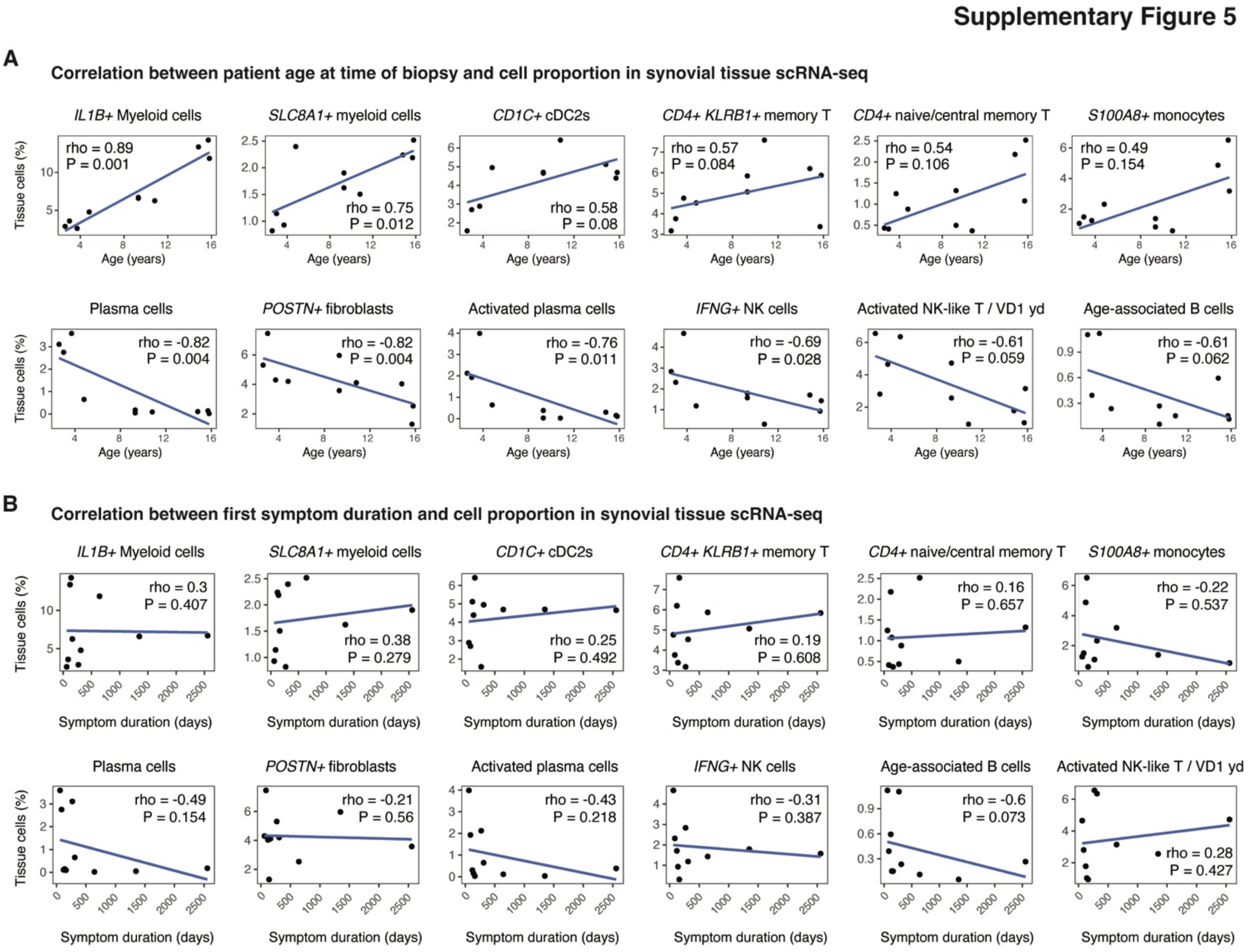

Figure S5: **The impact of age on cell type proportions in the inflamed joint of children with JIA.** (**A**) Correlation between participant age at time of biopsy and cell type proportions in the synovial tissue of children with JIA (scRNA-seq, *n* = 10). The 6 cell states with the strongest positive and negative correlations are shown (Spearman's rho, unadjusted *P* values). Each dot represents a sample. (**B**) Correlation between symptom duration and cell type proportions in the synovial tissue of children with JIA for the cell states shown in (**A**) (Spearman's rho, unadjusted *P* values). Each dot represents a sample.

Supplementary Figure 6

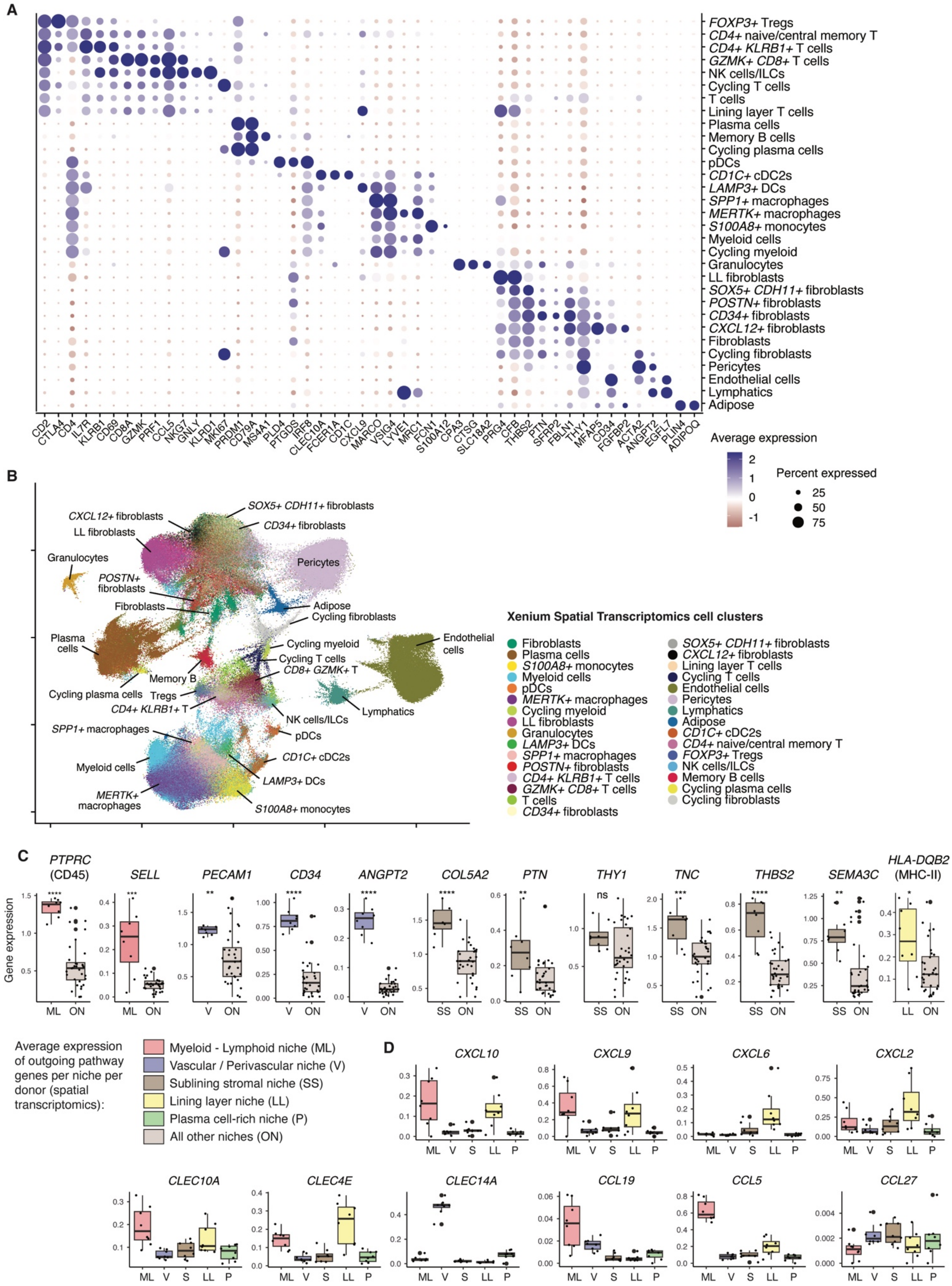

Figure S6: **Identification and localization of fine cell states from spatial transcriptomics analysis.** (**A**) Key markers of cell states identified from spatial transcriptomic analysis of synovial tissue (*n* = 8, 365,279 cells over 413 slides) at high resolution clustering. (**B**) UMAP embedding of cell states identified in spatial transcriptomics. Samples as per (**A**). (**C-D**) Average expression of genes, from predicted outgoing signaling pathways of **Fig. 4D**, in cells belonging to different niches that (**C**) confirmed the findings or (**D**) did not demonstrate enrichment in the same niches. Box plots show median gene expression from spatial transcriptomics according to identified niches (*n* = 8). Individual dots = average gene expression across cells, per niche, for each donor. Box plots show median, IQR and highest/lowest value within 1.5 * IQR. X axis abbreviations as indicated in color key. In (**C**) Unadjusted P values * ≤ 0.05; ** ≤ 0.01; *** ≤ 0.001; **** ≤ 0.0001, Wilcoxon rank sum test.

Supplementary Figure 7

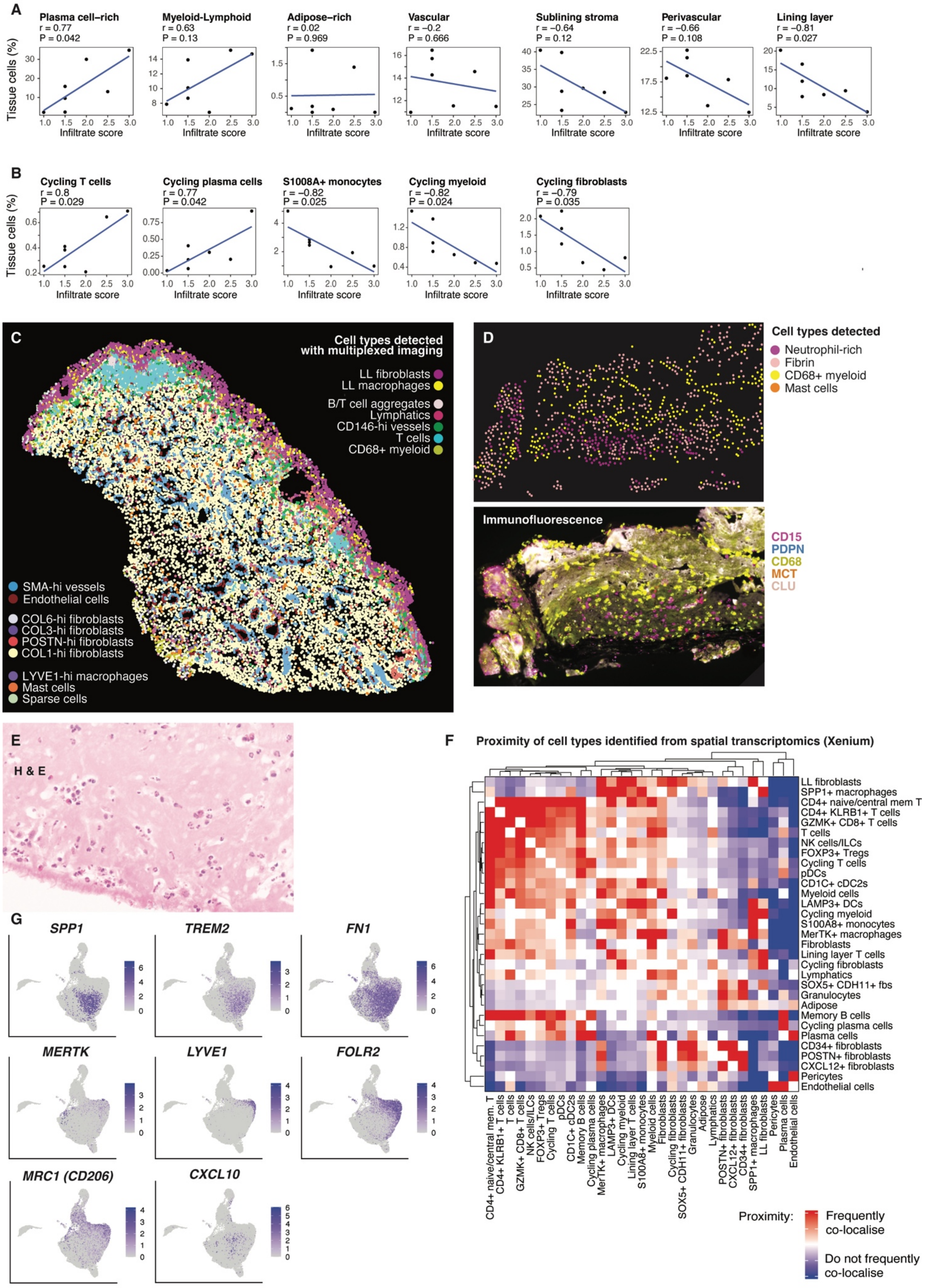

Figure S7: **Validation of cellular localization and signaling within niches using a multi-modal approach.** (**A-B**) Correlation between proportions from spatial transcriptomics of (**A**) each niche or (**B**) each cell type, and Krenn inflammatory infiltrate scores across each sample, $n = 7$. Pearson's correlation coefficient (r) and unadjusted P values as indicated. Krenn infiltrate scoring based on H&E sections from the same biopsies, as scored by at least two histopathologists (average score shown). Each dot represents a donor. In (**B**), cell types correlated with P value < 0.05 shown. (**C**) Cell type detection of full biopsy fragment imaged with multiplexed immunofluorescence (Leica Cell DIVE) shown in **Fig 5D**. (**D**) Representative example showing the infiltration of CD15+ neutrophils and CD68+ myeloid cells in fibrin deposits on multiplexed imaging (lower panel) and the corresponding cell annotation (upper panel). (**E**) Representative example of multi-lobed neutrophils infiltrating fibrin deposits from H&E. (**F**) Proximity analysis of cells from spatial transcriptomics based on the nearest neighbor of each cell type with hierarchical clustering identifies groups of cells approximating the same cell types, $n = 8$. Color scale, frequency of co-localization. (**G**) Feature plot of residency markers across myeloid cells from tissue scRNA-seq samples ($n = 10$).

Supplementary Figure 8

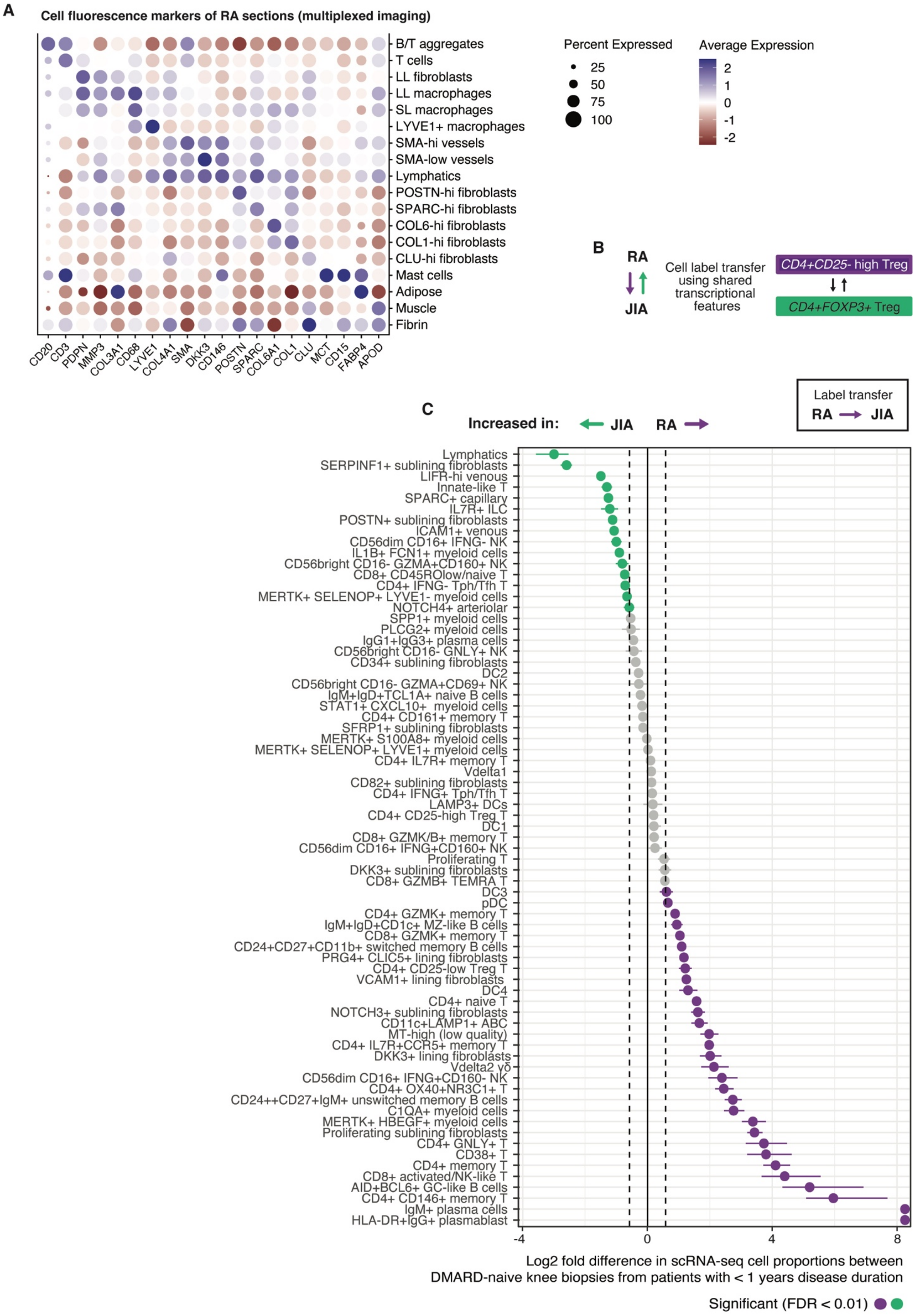

Figure S8: **Synovial tissue from children with JIA shows key distinctions to biopsies from adults with RA when matched for disease duration and site.** (**A**) Dotplot of average expression of immunofluorescence markers detected per cell type following integration, clustering and manual annotation of n = 6 biopsies from adults with RA and a disease duration < 1.06 years, visualized by multiplexed immunofluorescence. (**B**) Schematic of cell label transfer analysis to identify corresponding cell states from scRNA-seq datasets of synovial tissue from children with JIA (n = 10) and adults with RA (n = 69). (**C**) Projecting scRNA-seq cluster labels from knee biopsies of adults with RA (Zhang et al 2023, AMP2 Consortium, n = 12 individuals) onto our JIA dataset identifies cell states proportionally enriched in the different cohorts. Label transfer is performed in the opposite direction to that shown in **Fig 7G**, (FDR < 0.01, > 0.58 log2 fold difference, 10,000 permutations). Non-significant enrichments shown in grey. Cell states shown that were present with >200 cells in pooled data.

Supplementary Figure 9

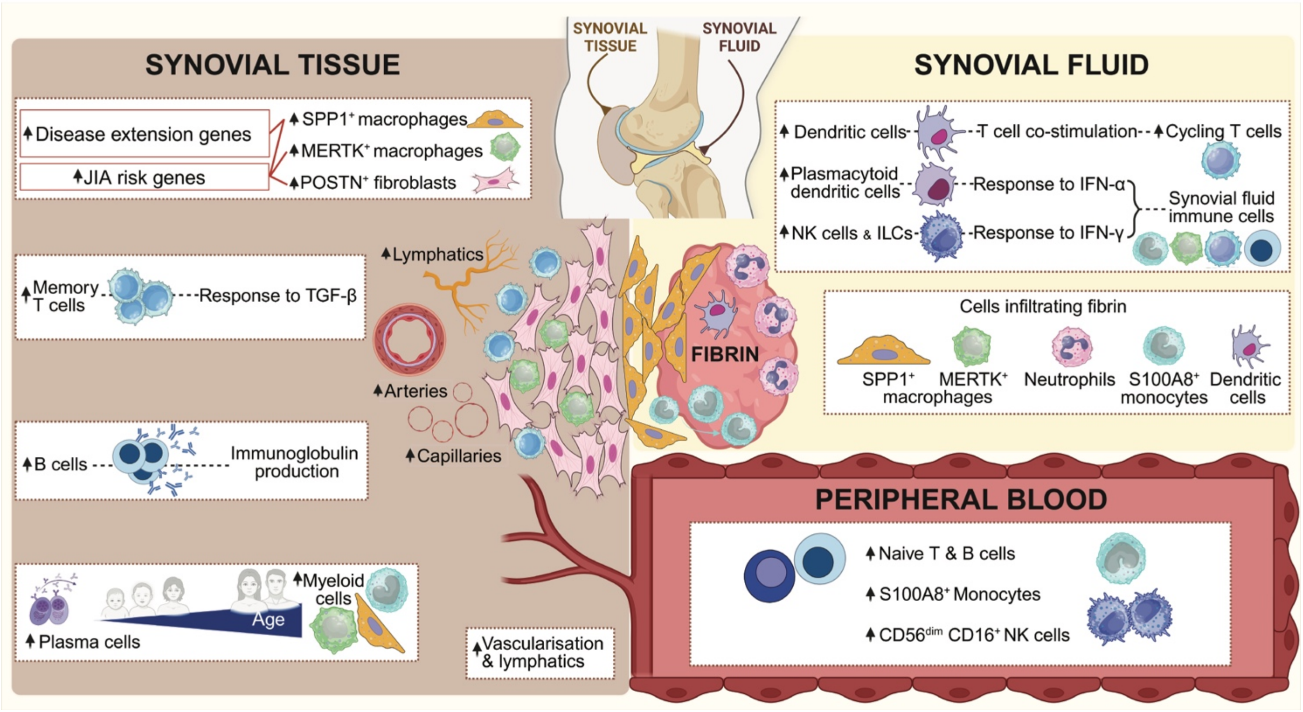

Figure S9: The synovial cavity exhibits distinct features of immune dysregulation compared to synovial tissue. Infographic summarizing differences in inferred signaling pathways and cellular enrichment of different anatomical compartments from analyses in **Fig 2** & **Fig 3**. Created in BioRender. Neag, G. (2025) https://BioRender.com/mogme60.

Supplementary Figure 10

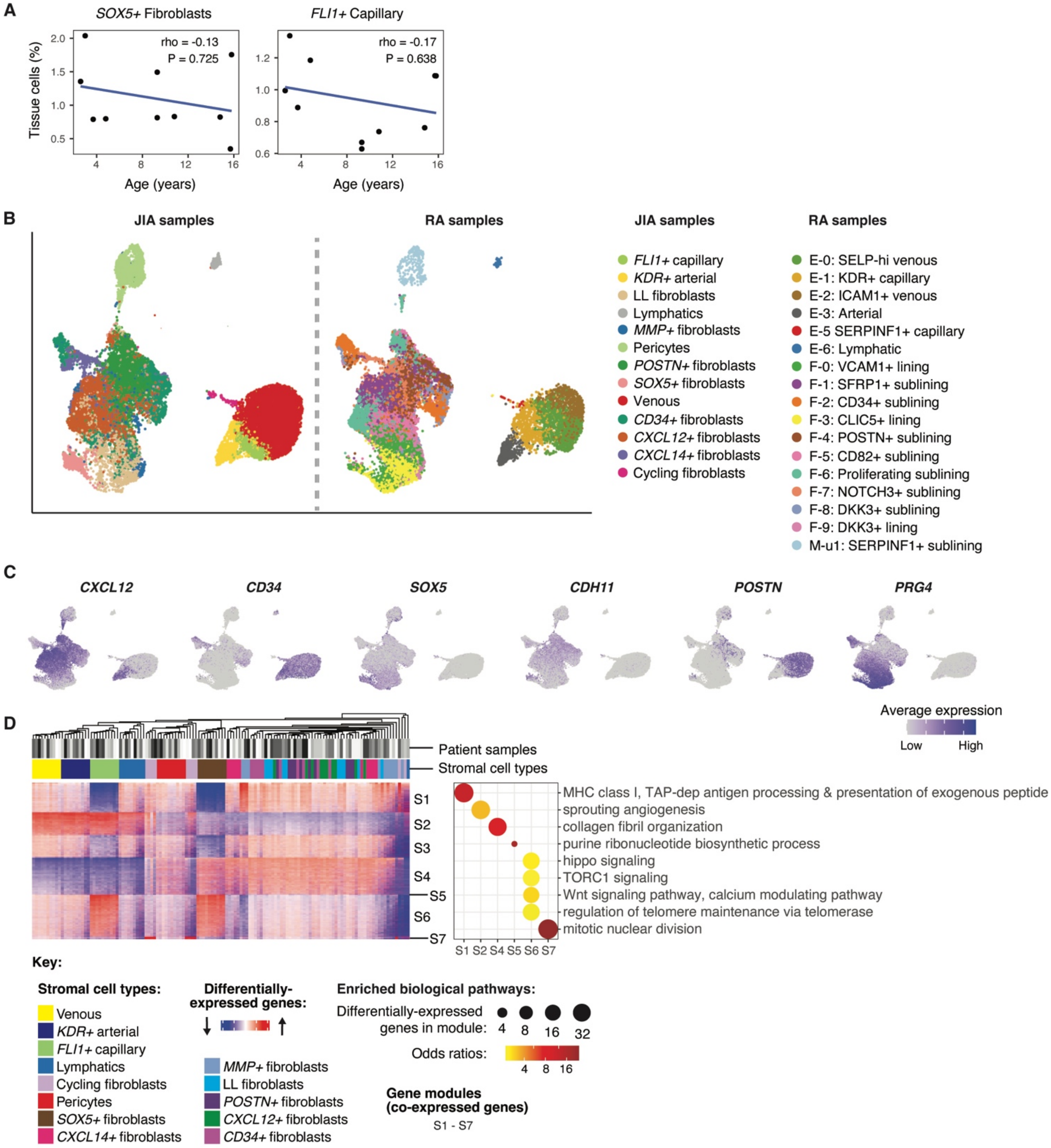

Figure S10: ***SOX5*+ fibroblasts and *FLI1*+ capillary cells share upregulated gene expression programs.** (**A**) Correlation between patient age and proportion of *SOX5+* fibroblasts and *FLI1+* capillary cells in synovial tissue scRNA-seq from participants with JIA (Spearman's rho, unadjusted *P* values). Each dot represents a sample, *n* = 10. (**B**) Original annotation of stromal cell

types from JIA (*n* = 7, left) and RA (*n* = 12, right) synovial tissue samples following integration of scRNA-seq data. (**C**) Feature plots showing example gene markers for the integrated stromal clusters showing expression of lining layer markers (*PRG4*) and sublining markers (*CDH11, POSTN, CD34*) within the *SOX5*+ fibroblast cluster, total *n* = 19 from JIA and RA samples. (**D**) Pseudobulk analysis of stromal cells from JIA scRNA-seq dataset, *n* = 10. Left: heatmap showing modules of co-expressed genes; right: dotplot showing biological pathway analysis.

Table S1: Demographics, clinical and histology details for the pediatric JIA cohort.

Table S2: Global marker genes (scRNA-seq).

Table S3: Pseudobulk analysis of differentially expressed genes for the main lineages (macrophages, T cells, B cells, dendritic cells; from scRNA-seq).

Table S4: Gene ontology terms for the main cell lineages in PBMC (macrophages, T cell, B cells and dendritic cells).

Table S5: Top 20 gene markers for the fine subclusters of each cell lineage (scRNA-seq).

Table S6: Top 30 biological pathways per stromal cluster based on differential gene expression (scRNA-seq).

Table S7: Spatial transcriptomics panel probe list.

Table S8: Markers of fine subclusters (spatial transcriptomics).

Table S9: Immunofluorescence antibodies used for multiplexed immunofluorescence (Leica Cell DIVE) and confocal microscopy.

Table S10: Demographics, clinical and histology details for RA cohort (scRNA-seq)

Table S11: Demographics, clinical and histology details for the adult RA cohort (multiplexed immunofluorescence

Table S12: Differentially expressed genes from bulk RNA-sequencing of TGF-b stimulated synovial fibroblasts compared to vehicle control.
